# The role of glacial‐interglacial climate change in shaping the genetic structure of eastern subterranean termites in the southern Appalachian Mountains, USA

**DOI:** 10.1002/ece3.5065

**Published:** 2019-04-01

**Authors:** Chaz Hyseni, Ryan C. Garrick

**Affiliations:** ^1^ Department of Biology University of Mississippi Oxford Mississippi

**Keywords:** approximate Bayesian computation, demographic history, distributional shift, evolutionary history, machine learning, phylogeography, range contraction, range expansion, *Reticulitermes flavipes*, species distribution modeling

## Abstract

The eastern subterranean termite, *Reticulitermes flavipes*, currently inhabits previously glaciated regions of the northeastern U.S., as well as the unglaciated southern Appalachian Mountains and surrounding areas. We hypothesized that Pleistocene climatic fluctuations have influenced the distribution of *R. flavipes*, and thus the evolutionary history of the species. We estimated contemporary and historical geographic distributions of *R. flavipes* by constructing Species Distribution Models (SDM). We also inferred the evolutionary and demographic history of the species using mitochondrial (cytochrome oxidase I and II) and nuclear (endo‐beta‐1,4‐glucanase) DNA sequence data. To do this, genetic populations were delineated using Bayesian spatial‐genetic clustering, competing hypotheses about population divergence were assessed using approximate Bayesian computation (ABC), and changes in population size were estimated using Bayesian skyline plots. SDMs identified areas in the north with suitable habitat during the transition from the Last Interglacial to the Last Glacial Maximum, as well as an expanding distribution from the mid‐Holocene to the present. Genetic analyses identified three geographically cohesive populations, corresponding with northern, central, and southern portions of the study region. Based on ABC analyses, divergence between the Northern and Southern populations was the oldest, estimated to have occurred 64.80 thousand years ago (kya), which corresponds with the timing of available habitat in the north. The Central and Northern populations diverged in the mid‐Holocene, 8.63 kya, after which the Central population continued to expand. Accordingly, phylogeographic patterns of *R. flavipes* in the southern Appalachians appear to have been strongly influenced by glacial‐interglacial climate change.

**OPEN RESEARCH BADGES:**

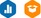

This article has been awarded Open Materials, Open Data Badges. All materials and data are publicly accessible via the Open Science Framework at https://doi.org/10.5061/dryad.5hr7f31.

## 1 INTRODUCTION

Geographic barriers to dispersal, such as mountains and rivers, are considered major drivers of genetic divergence within and among species. The influence of climate change (e.g., glacial‐interglacial oscillations during the Pleistocene) in generating phylogeographic structure is also widely recognized (Hewitt, 1996; Avise, 2000 and references therein). For example, in Europe, when ice sheets reached their maximum extent during glacials, this repeatedly resulted in range contraction into southern refugia, which subsequently served as key reservoirs for recolonization via northward expansion during interglacials (Hewitt, 1996,2004). In these regions at high latitudes, successive glacial‐interglacial cycles were likely to reinforce the same genetic signatures of contraction and expansion (but see Gomez & Lunt, 2007; Shafer, Cullingham, Cote, & Coltman, 2010).

In contrast to landscapes that were repeatedly covered by ice sheet advances throughout the Pleistocene, those in temperate or tropical regions that remained unglaciated potentially contained numerous refugia (Byrne, 2008). Indeed, in montane areas with deeply dissected topography, latitude alone may be a poor proxy for the locations of refugial areas, as the steep environmental gradients that occur locally can exert a strong influence on persistence of habitat patches that can support viable populations. In such regions—in contrast to the traditional view of refuges being continuously occupied long‐term stable areas—successive glacial‐interglacial cycles are less likely to have repeatedly played out in the same way. Owing to stochastic processes, they may have instead been somewhat ephemeral. For instance, a refugium may have been only periodically occupied, with the process of shifting between alternative refugia from one glacial cycle to the next involving extinction at the trailing edge and colonization at the leading edge. Herein, we refer to this particular case of contraction–expansion dynamics as “distributional shift” and consider it a plausible model for the focal landscape setting. Indeed, consideration of how major shifts in geographic distributions contributed to population differentiation during the Pleistocene is important for understanding speciation processes (Carstens & Knowles, 2007 and references therein).

The southern Appalachian Mountains represent some of the oldest uplands in North America (~471–480 million years old; Hibbard, Meehl, Cox, & Friedlingstein, 2007 and references therein) and harbor high levels of biodiversity (Crandall & Buhay, 2008; Marek & Bond, 2009; Petranka, 1998; Rissler & Smith, 2010). This topographically complex temperate region is characterized by steep environmental gradients, which have promoted population divergence in many species, particularly those with poor dispersal abilities (Hedin & Wood, 2002). Paleoclimatic (Loehle, 2007), biogeographic (Swenson & Howard, 2005) and comparative phylogeographic (Soltis, Morris, McLachlan, Manos, & Soltis, 2006) data indicate that the southern Appalachians remained free from Pleistocene ice sheet advances, and consequently, retained numerous refugial areas for forest‐dependent biota during cool and dry glacial periods. Indeed, short‐range endemism and high diversity have been well documented in plethodontid salamanders (Petranka, 1998) and other amphibians (Rissler & Smith, 2010). Similar patterns have also been reported for invertebrate groups such as crayfish (Crandall & Buhay, 2008), arachnids (Hedin & Wood, 2002; Thomas & Hedin, 2008), and millipedes (Marek, 2010). While the role of the southern Appalachian Mountains as a major barrier driving an east‐west divide among lowland taxa is widely recognized (Soltis et al., 2006 and references therein), there have been surprisingly few biogeographic and phylogeographic studies of upland species that occupy the mid‐ and high‐elevation ridgelines, and research on invertebrates, in particular, is underrepresented.

The eastern subterranean termite, *Reticulitermes flavipes*, currently inhabits previously glaciated regions of the northeastern U.S., as well as the unglaciated southern Appalachian Mountains and surrounding areas. This species is a key ecosystem engineer that makes major contributions to dead‐wood decomposition and nutrient cycling in forests (Myer & Forschler, 2019; Ulyshen, Wagner, & Mulrooney, 2014), and its distribution is influenced by humidity and temperature (Wiltz, 2015). This diploid eusocial species live in colonies that typically have a simple family structure, arising from an outbred primary reproductive pair that remains fertile for 6–11 years (Vargo & Husseneder, 2009). When the king or queen dies, some full‐sib workers differentiate into male and female secondary reproductives, at which point the colony becomes inbred (Vargo & Carlson, 2006). However, in addition to temporal transitions from simple to extended families, there may also be spatial partitioning, whereby the initial reproductive center, with the primary reproductives, expands into satellite nests housing secondary reproductives (Thorne, Traniello, Adams, & Bulmer, 1999). Winged alates disperse away from the original colony and establish new colonies and then shed their wings. However, dispersal abilities are only moderate, with distances varying from a few meters to >1 km (Vargo & Husseneder, 2009). Such limited dispersal is conducive to strong historical inference (Cruzan & Templeton, 2000).

Reconstructing long‐term population history is often achieved via analyses of geo‐referenced DNA sequence data, using spatially explicit phylogenetic and/or coalescent‐based analytical approaches (see Knowles, 2009; Hickerson et al., 2010 and references therein). Increasingly, complementary nongenetic data are being employed to augment inferences or to generate hypotheses about past events and population processes. In particular, Species Distribution Models (SDM) are now widely used to locate glacial refugia (Richards, Carstens, & Knowles, 2007), or determine the influence of past climate change on current genetic structure (Alexandrino, Teixeira, Arntzen, & Ferrand, 2007). In some cases, similar conclusions about phylogeographic history have been drawn from SDMs and genetic data (Waltari et al., 2007). Briefly, SDMs relate occurrence records for a given species with the environmental conditions in those same locations in order to estimate geographic areas in which the species is likely to be found (Guisan & Thuiller, 2005). Given that historical climatic fluctuations can trigger range contractions and expansions—including wholesale distributional shifts (Pielou, 1991)—SDMs can form a framework for understanding the genetic consequences of glacial‐interglacial climate change (Knowles & Alvarado‐Serrano, 2010).

In this study, we investigated the genetic consequences of glacial‐interglacial climate change on *R. flavipes* from the unglaciated southern Appalachian Mountains and surrounding areas, and considered distributional shifts as a plausible hypothesis (among others) to be assessed using SDMs and genetic data. Given the reliance of this species on dead‐wood microhabitats, our expectation was that during the Pleistocene and earlier, *R. flavipes* closely tracked the changing distributions of forest habitats, and was strongly impacted by climatic fluctuations. Indeed, ecologically specialized low‐mobility forest insects may be particularly well‐suited for reconstructing past climatic impacts on montane forest landscapes, in part owing to their short generation times and ability to persist in habitat patches too small to support more mobile vertebrates (Garrick et al., 2004; Hugall, Moritz, Moussalli, & Stanisic, 2002; Sunnucks et al., 2006). Furthermore, owing to the limited dispersal ability of *R. flavipes*, we expected that relatively fine‐scale genetic structuring would be detectable. To test these expectations, we modeled present and past distributions and used contrasts between these SDMs to make inferences about distributional shifts and to identify areas of stability (i.e., potential refugia). Based on this, we generated competing hypotheses about drivers of genetic divergence, and then tested these via analyses of DNA sequence data using coalescent simulations. In addition to the effects of historical climatic conditions, we also considered the influence, if any, of contemporary climatic conditions and dispersal‐based spatial structure on genetic variation in *R. flavipes*.

## 2 METHODS

### 2.1 Phylogeographic framework

To address the aims of this study, we used the following workflow:

Step 1—Model present‐day and historical climate‐based distributions of *R. flavipes* in order to identify potential refugia and generate expectations about directionality of range contractions or expansions, including distributional shifts;
Step 2—Infer the number of distinct populations using spatial‐genetic clustering, and cross‐validate via principal component analysis, and phylogenetic reconstruction; characterize genetic variation within and differentiation among populations, and; estimate the amount of genetic variation explained by dispersal (spatial structure) and environment (contemporary climatic conditions);
Step 3—Test alternative phylogeographic hypotheses to determine whether expansion out of refugia, distributional shifts, or vicariance was the underlying historical process generating the observed patterns of genetic variation within and among populations; estimate values of parameters included in the best‐fit phylogeographic hypothesis; and assess evidence for changes in effective population size over time.

### 2.2 Genetic data collection

**Figure 1:**
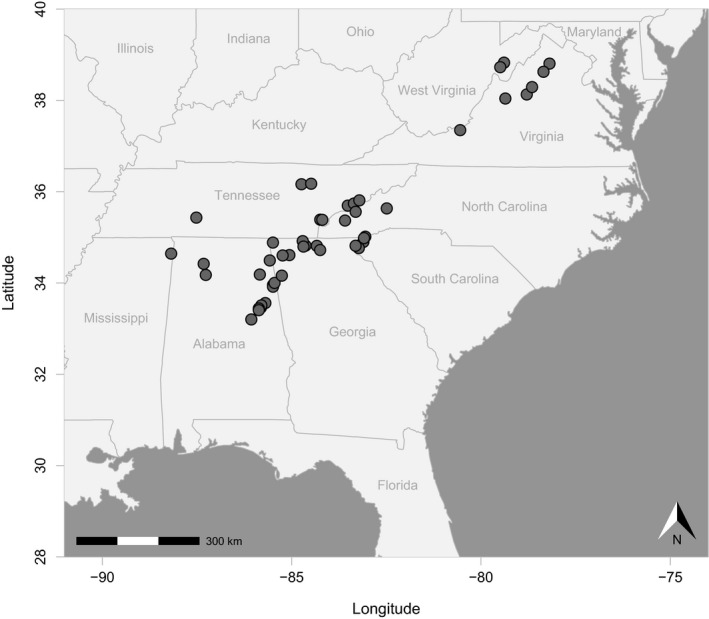
Sites sampled for use in genetic analyses. Geographic map showing sampling locations (gray dots, *n* = 46) from which *Reticulitermes flavipes* termites were collected in the southern Appalachian Mountains, southeastern USA

*Reticulitermes* termites were collected between 2012 and 2014 from locations in the southern Appalachian Mountains. Since it is not possible to reliably distinguish among several co‐distributed species on the basis of morphology when only members of the worker caste are collected (Wang et al., 2009), termites were identified using a molecular assay (Garrick, Collins, Yi, Dyer, & Hyseni, 2015). Ultimately, *R. flavipes* were sampled from 50 rotting logs across 46 locations (Figure 1; also see Supporting Information Table S1.1). From each log, 1–3 individuals were used for phylogeographic analyses. For out‐group taxa, we included specimens representing three close relatives (Supporting Information Table S1.2): *R. virginicus* (*n* = 3 individuals), *R. malletei* (*n* = 1) and *R. nelsonae* (*n* = 1).

Extraction of genomic DNA was performed using a DNeasy tissue kit (Qiagen, Valencia, CA) following the manufacturer's recommendations. Portions of the mitochondrial cytochrome c oxidase subunit I (COI) and II (COII) genes, and an intronic portion of the nuclear endo‐beta‐1,4‐glucanase (EB14G) gene, were amplified via polymerase chain reaction using primers (Supporting Information Table S2.3) and conditions reported in Supporting Information Appendix S2, and then sequenced at Yale University. Sequence alignments were performed using Geneious v.6.1.8 (Kearse et al., 2012), and manually edited as necessary.

We concatenated COI and COII and refer to this sequence (COI + COII) as the mitochondrial DNA (mtDNA) locus; we refer to EB14G as the nuclear DNA (nDNA) locus. For the latter, heterozygous sites were scored using the “Find Heterozygotes” plugin in Geneious. For a site to be considered heterozygous, we required that height of the secondary peak was at least 50% of the primary peak (sites with quality scores < 20, were coded as “N”). Allele haplotypes were inferred using PHASE v.2.1.1 (Stephens, Smith, & Donnelly, 2001), with the following settings: 90% phase certainty, 10,000 iterations, thinning interval = 10, burn‐in = 1,000, and the default recombination model. PHASE was run three times to evaluate consistency of results.

### 2.3 Step 1: Present and past geographic distributions

**Figure 2:**
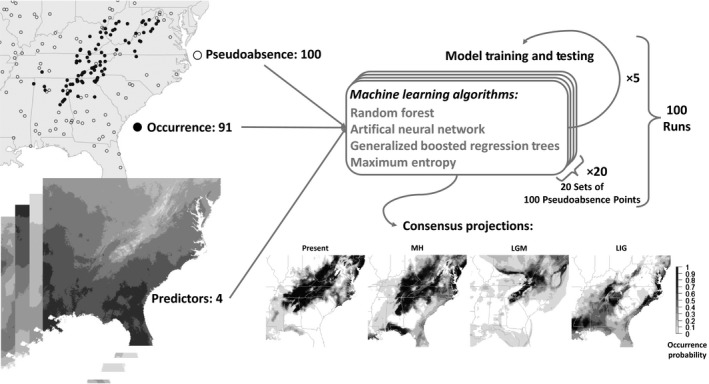
Species Distribution Modeling. Diagram showing the conceptual framework used to generate SDMs that enabled contrasts between successive time periods: “present” (1960–1990), Mid‐Holocene (MH; ~6 kya), Last Glacial Maximum (LGM; ~22 kya), and Last Interglacial (LIG; ~120–140 kya)

There are few published occurrence records of forest populations of *R. flavipes* with confirmed species‐level identifications and adequate geospatial precision for SDM. Accordingly, in addition to the 46 sites that contributed to genetic analyses (above), the presence of *R. flavipes* at an additional 45 locations (surveyed from 2015 to 2016) was confirmed using Garrick et al.'s (2015) molecular assay, resulting in a total of 91 occurrence points (Supporting Information Table S1.1). To construct SDMs, we used the “biomod2” package (Thuiller, Georges, Engler, & Breiner, 2016) in R (R Core Team, 2018). Full details about SDM construction are given in Supporting Information Appendix S3. Briefly, we used four machine learning algorithms to model distributions based on climatological data, presence records, and 20 independent sets of 100 pseudo‐absence points (Figure 2). The latter choice was based on work by Barbet‐Massin, Jiguet, Albert, and Thuiller (2012), who showed that for machine learning methods it is better to use multiple replicates of pseudo‐absence points, with the number of pseudo‐absences in each replicate close to the number of occurrence points. We used environmental variables at a 1‐km resolution for SDM construction. Present‐day SDMs were based on mean climatological data spanning 1960–1990, and historical distributions were modeled for the Mid‐Holocene (MH; ~6 kya), the Last Glacial Maximum (LGM, ~22 kya), and the Last Interglacial (LIG, ~120–140 kya). For each period, 19 bioclimatic variables (Hijmans, Cameron, Parra, Jones, & Jarvis, 2005) were obtained from the WorldClim database v.1.4 (http://www.worldclim.org; Supporting Information Table S3.4), and then factor analysis was used in order to retain maximum variation contained in the 19 bioclimatic variables while simultaneously: (a) reducing the number of predictors, to avoid overfitting, and (b) dealing with nonindependence of predictors (i.e., collinearity), which represents a serious challenge to most correlative modeling methods (Dormann et al., 2013).

#### 2.3.1 Distributional shifts and areas of stability

We used a threshold value to convert continuous occurrence probabilities to a binary classification of suitable (>0.2) versus unsuitable (≤0.2). The occurrence probability threshold was chosen based on the True Skill Statistic (TSS; Allouche, Tsoar, & Kadmon, 2006). Specifically, we chose a threshold value that maximized the TSS, as this approach has consistently performed better than other thresholding methods (Liu, Berry, Dawson, & Pearson, 2005; Liu, Newell, & White, 2016; Liu, White, & Newell, 2013). However, since we used multiple pseudo‐absence replicates, we had the opportunity to maximize TSS without risking under‐prediction of presences, which results from choosing a high threshold value. Indeed, using distributions of TSS and threshold values, we were able to select the lowest threshold (0.2; Supporting Information Figure S3.1), below which TSS had a steep slope. To calculate the distributional shift between two successive time periods (e.g., LIG to LGM, or LGM to MH), we took the difference of the two binary maps, after multiplying the more recent time period by two in order to ensure that we obtain four categories in the distributional shift calculation: colonization (difference = 2), stability (1), absence (0), and extinction (−1; see Supporting Information Figure S3.2). Similarly, to estimate areas of stability (i.e., persistence in a location between successive time periods), we multiplied the binary occurrence maps (Supporting Information Figure S3.2) of the corresponding periods: locations where the product is 1 were considered to harbor stable habitats across time periods (stability = 1).

### 2.4 Step 2: Genetic variation and the role of environment and space in genetic structuring

#### 2.4.1 Bayesian clustering and Principal Components Analysis

To determine the number of geographically cohesive genetic groups of *R. flavipes*, we analyzed geo‐referenced mtDNA sequences in BAPS v.6.0 (Cheng, Connor, Siren, Aanensen, & Corander, 2013). We assessed values of *K* (i.e., the number of clusters) ranging from 2–20, with 10 replicate runs each. We also examined evidence for geographically cohesive genetic groups by representing the variance in mtDNA sequences using Principal Components Analysis (PCA), performed with the “prcomp” function in R.

#### 2.4.2 Phylogenetic reconstruction and molecular dating

We reconstructed a mtDNA‐based dated phylogeny to verify the existence of any genetic groups determined by BAPS, as well as to estimate divergence times. First, we used PartitionFinder 1.1.0 (Lanfear, Calcott, Ho, & Guindon, 2012) to determine the best partitioning scheme, and the Bayesian Information Criterion in jModelTest v.2.1.10 (Darriba, Taboada, Doallo, & Posada, 2012) to identify the optimal model of sequence evolution. The best‐fit model for all three codon positions was HKY + I (Hasegawa, Kishino, & Yano, 1985). Then, to estimate a dated phylogeny, we used BEAST v.2.4.5 (Bouckaert et al., 2014), with a relaxed log‐normal molecular clock (Drummond, Ho, Phillips, & Rambaut, 2006), and a coalescent tree prior. We used broad mutation rate priors. For the mtDNA locus, the range included Brower's (1994) commonly used insect rate of 1.15% sequence divergence per lineage per million years, and Luchetti, Marini, and Mantovani (2005) faster rate of up to 140% per million years, which was estimated from COII in European *Reticulitermes* taxa. Based on point estimates obtained using approximate Bayesian computation (ABC; Beaumont, Zhang, & Balding, 2002) assessments of competing phylogeographic hypotheses (described in Section 2—Step 3), we set the mean mutation rate at 12% per million years (see Section 3—Step 3) for the mtDNA locus. Since there was no mutation rate information available for the nDNA locus in *Reticulitermes*, we estimated the mean mutation rate in BEAST by conditioning on the mtDNA locus and setting the initial mean value at 0.6% with a range of 0.2%–2% (obtained using ABC; see Section 3—Step 3). BEAST was run for 50 million Markov chain Monte Carlo generations, with samples saved every 2,500 generations, after discarding the first 5 million generations as burn‐in. We used Tracer 1.6 (Rambaut, Drummond, Xie, Baele, & Suchard, 2018) to examine the stationarity of parameter estimates and to determine that effective sample sizes were greater than 500. BEAST was run with and without the out‐group *Reticulitermes* taxa using the same settings. Results were summarized via a Maximum Clade Credibility tree in TreeAnnotator v.2.4.4 (Bouckaert et al., 2014), with the first 25% of trees discarded as burn‐in.

#### 2.4.3 Diversity within and differentiation among genetic populations

To estimate levels of diversity within each genetic population, the following metrics were calculated separately for the mtDNA and nDNA loci using DnaSP v5.10.01 (Librado & Rozas, 2009): number of segregating sites (*S*; Nei, 1987), average number of nucleotide differences (*K*; Tajima, 1983), nucleotide diversity (*π*; Nei, 1987), and the mutation‐scaled effective population size (*θ*_W_; Watterson, 1975). To measure genetic divergence among genetic populations, the following statistics were also calculated: average number of nucleotide substitutions per site (Dxy; Nei, 1987), net number of nucleotide substitutions per site (Da; Nei, 1987), average number of pairwise nucleotide differences (Kxy; Tajima, 1983), and *F*_ST_ (Hudson, Slatkin, & Maddison, 1992).

#### 2.4.4 Genetic variation influenced by environment and dispersal

To estimate the amount of genetic variation explained by spatial structure versus the environment, we used distance‐based redundancy analysis (dbRDA; Legendre & Anderson, 1999). We computed the genetic distance matrix using the “dist.dna” function of the “ape” (Paradis & Schliep, 2019) package, and performed dbRDA using the “capscale” function of the “vegan” (Oksanen et al., 2018) package in R. To compute the response variable, genetic distances (i.e., matrix of pairwise mutational differences between DNA sequences) were estimated using the TN93 (Tamura & Nei, 1993) model of sequence evolution, allowing for different rates for transitions and transversions. For environmental predictors, we used the contemporary environmental factors obtained via factor analysis (see Section 2—Step 1). To obtain spatial structure predictors, we transformed Euclidean geographic distances to a continuous rectangular vector by Principal Coordinates analysis of Neighbor Matrices (PCNM) using the “pcnm” function in “vegan.” Significance of the predictors was assessed using multivariate *F*‐statistics with 9,999 permutations. We first analyzed the relationship between the genetic distance matrix and each environmental factor separately, and then performed a partial dbRDA for each variable while controlling for the influence of spatial structure, using only significant PCNM eigenvectors. Similarly, we analyzed the relationship between genetic distances and PCNM eigenvectors, retained the significant eigenvectors, and then removed interactions with the environment to obtain the contribution of spatial structure alone.

### 2.5 Step 3: Phylogeographic hypothesis testing and population size changes

#### 2.5.1 Competing scenarios

We used ABC, as implemented in the software DIYABC v.2.1.0 (Cornuet et al., 2014), to assess alternative hypotheses designed to determine whether expansion out of long‐term stable refugia, distributional shifts, or vicariance was the major underlying process generating the present‐day spatial distribution of genetic variation. mtDNA plus (phased) nDNA sequence data were used, and we conditioned these analyses on a posteriori knowledge of the existence of three distinct genetic clusters of *R. flavipes* (see Section 3—Step 2). Because ABC analyses can suffer when a large number of candidate models are simultaneously considered (Pelletier & Carstens, 2014), we employed a two‐tiered approach, where best‐fit scenarios from separate analyses in the first tier are subsequently compared against each other in the second tier. This hierarchical or tournament‐style approach has also been applied in other study systems (Espindola et al., 2016; Stone et al., 2017).

All scenarios in both tiers incorporated bottleneck events, because they all involved divergence of new populations from an existing population, and thus founder effects. Indeed, our inclusion of bottleneck events enabled specification of progenitor‐descendant relationships between pairs of diverging populations (as in Garrick et al., 2014). Furthermore, the nonnegligible role of bottlenecks during climatically driven population divergence has been established. In one set of analyses in the first tier of ABC comparisons, we assessed scenarios in which *R. flavipes* persisted in a single major refugium (Supporting Information Figure S4.3)*,* such that the other areas were colonized via successive expansions out of that refugium. We considered three different refugial locations (i.e., the north, south, or central portion of the study region; see Section 3—Step 2). In a second set of analyses within the first tier, we assessed scenarios that involved distributional shifts (Supporting Information Figure S4.4), whereby populations diverged in a stepping‐stone fashion (i.e., one population gave rise to a descendant population, which later became the progenitor of the third population). Here, we considered all possible stepping‐stone configurations (i.e., there was no assumption that only nearest neighbors can exhibit a progenitor‐descendant relationship). In the second tier of ABC comparisons, the best‐fit hypotheses from the refugial and distributional shift scenarios were directly compared, along with an additional hypothesis that incorporated vicariance (Supporting Information Figure S4.5). The reason for including this third hypothesis was to test the possibility that the original ancestral population no longer exists, having split into two new populations, one of them giving rise to a third population. While there are other vicariance hypotheses that could have been compared in the first tier, we chose not to do this based on the sequence of divergence events best‐fit refugial and distributional shift hypotheses had in common. This reduced the number of plausible vicariance hypotheses to one.

#### 2.5.2 ABC model specification, and model choice

Within the ABC framework, two classes of model parameters were used to characterize the phylogeographic hypotheses described above: effective population sizes (Ne), and divergence times (*T*). We performed two rounds of modeling: (a) a preliminary round with broad priors, and (b) the final round with narrower priors (Supporting Information Table S4.5). Briefly, all competing scenarios had two divergence events: any two of *T*_N_, *T*_C_ or *T*_S, _(where the subscript is the first letter abbreviation of the new cluster, i.e., Northern, Central, or Southern), the prior range for the more recent event encompassed the MH and the LGM whereas priors for the older event ranged from the LGM to the LIG assuming a 1‐year generation time for *R. flavipes*. Full details of ABC priors on Ne and T parameters are given in Supporting Information Appendix S4. We set the mtDNA mutation rate priors from 5.0 × 10^−9^ to 5.0 × 10^−7^, a broad range encompassing the Brower (1994) and Luchetti et al. (2005) rates (see Section 2—Step 2). Similarly, since no rates were available for the nDNA locus in *Reticulitermes*, we used broad priors for this locus, from 5.0 × 10^−10^ to 2.5 × 10^−8^. Thus, the mean nDNA rate was an order of magnitude slower than the mean mtDNA rate, despite some overlap at the upper end of nDNA and lower end of mtDNA prior ranges.

To characterize the empirical two‐locus DNA sequence dataset, we used the following summary statistics: number of segregating sites (one‐ and two‐sample) and private segregating sites (one‐sample), mean (one‐ and two‐sample) and variance of pairwise differences (one‐sample), mean and variance of numbers of the rarest nucleotide at segregating sites (one‐sample), Tajima's (1989) *D* (one‐sample), and *F*_ST_ (Hudson et al., 1992) between two samples. ABC runs consisted of 1 × 10^6^ simulated genetic datasets per competing phylogeographic hypothesis. We then compared the values of summary statistics calculated from simulated datasets to those from the empirical dataset. Following Cornuet et al. (2014), model checking was performed via principal components analysis, and then posterior probabilities were calculated via logistic regression (Fagundes et al., 2007) on 1% of simulated data most similar to the empirical data, to identify the best‐fit model (Cornuet et al., 2008). We evaluated model performance (i.e., the ability to discriminate between the best‐supported and alternative scenarios), by estimating type I and type II error rates. To do this, we simulated 500 data sets and estimated the most likely model using a polychotomous logistic regression (Cornuet, Ravigne, & Estoup, 2010; Cornuet et al., 2008). The type I error rate was the proportion of data sets that were simulated under an alternative scenario but were incorrectly categorized under the best‐supported scenario. The type II error rate was the proportion of instances in which the best‐supported scenario was incorrectly selected as the most likely scenario. To calculate point estimates and confidence intervals for the values of parameters included in the best‐fit model, we selected 1% of the simulated data closest to the observed data. Additionally, for the best‐fit scenario, we estimated precision in parameter estimation (Cornuet et al., 2010) by computing the relative median of the absolute error for 500 simulated data sets with values drawn from posterior distributions.

#### 2.5.3 Population size changes over time

For each of the three *R. flavipes* genetic groups, we assessed evidence for population size changes versus stability by calculating Tajima's *D*, and Fu and Li's D* and F* (Fu & Li, 1993) from the mtDNA data, in DnaSP. To identify cases of departure from the null hypothesis of constant size, *p*‐values for these statistics were obtained by computing 10,000 coalescent simulations based on *θ* from the observed data and assuming no recombination. We also calculated Ramos‐Onsins and Rozas' (2002) *R* ^2^ statistic for which significantly small values indicate population growth, whereas significantly large *R* ^2^ values indicate size reduction. Statistical significance of deviation from the null hypothesis of constant population size was assessed by performing 10,000 coalescent simulations in DnaSP. To complement the above analyses, we also estimated mismatch distributions, where a unimodal distribution indicates growth, whereas a multimodal distribution is indicative of size constancy (Rogers & Harpending, 1992). Given that signatures of selection can mimic those of population size changes and therefore complicate interpretation of the above summary statistics, we examined evidence for nonneutrality using compound tests (Zeng, Shi, & Wu, 2007). We performed the compound tests using the program DH (http://zeng-lab.group.shef.ac.uk/wordpress). The significance (*α* = 0.05) of each test was determined using 100,000 simulations.

We also examined evidence for changes in Ne over time in each cluster by analyzing the combined mtDNA plus (unphased) nDNA sequence data using Extended Bayesian Skyline Plots (EBSP; Heled & Drummond, 2008) in BEAST. The same mutation rate parameters for phylogenetic tree estimation were used here, and EBSP searches were run for 50 million Markov chain Monte Carlo generations, with a burn‐in of 5 million generations. Samples were saved every 2,500 generations and ESS and the stationarity of likelihood values were examined in order to make sure all ESS values were greater than 500.

## 3 RESULTS

### 3.1 Genetic data collection

mtDNA sequences were obtained from 122 *R. flavipes* individuals, and the nDNA locus was sequenced from 124 individuals. The mtDNA alignment had 86 polymorphic sites and 32 haplotypes, while the nDNA locus had five polymorphic sites and five haplotypes (Table 1). All sampled logs contained individuals with the same mtDNA haplotype, with the exception of a rotting log sampled at site A41 (see Supporting Information Table S1.1), which contained two different haplotypes from the same genetic population, suggesting a rare instance of colony fusion (see DeHeer & Vargo, 2004).

**Table 1:** Genetic diversity and tests of neutrality

Data				Diversity						Neutrality		
DNA	Population	Locus	Individuals	No. of haplotypes	*S*	*π*	*θ*_Wnuc_	*K*	*θ*_Wgen_	Tajima*D*	FuLiD*	FuLiF*
mtDNA	Southern	COI	16	4	14	0.015	0.014	8.333	7.636	0.926	0.926	0.944
		COII		4	18	0.017	0.018	9.167	9.818	−0.678	−0.678	−0.700
		COI + COII		4	32	0.016	0.016	17.500	17.455	0.027	0.027	0.028
	Northern	COI	24	8	18	0.013	0.012	7.278	6.623	0.483	0.303	0.388
		COII		6	15	0.008	0.010	4.167	5.519	−1.182	−1.431	−1.535
		COI + COII		9	33	0.010	0.011	11.444	12.142	−0.289	−0.511	−0.513
	Central	COI	82	16	15	0.004	0.008	2.199	4.578	−1.957**	−2.270*	−2.527*
		COII		9	9	0.003	0.005	1.485	2.575	*−1.476#*	*−1.667#*	*−1.865#*
		COI + COII		19	24	0.003	0.006	3.684	7.153	−1.900**	−2.240*	−2.486*
	All	COI	122	28	46	0.014	0.021	7.823	11.671	−1.212	−0.754	−1.072
		COII		18	40	0.011	0.018	6.046	10.181	−1.482*	*−1.807#*	−2.008*
		COI + COII		32	86	0.012	0.020	13.869	21.851	−1.377	−1.316	−1.583
nDNA	All	EB14G	124	5	5	0.009	0.010	2.200	2.400	−0.562	−0.562	−0.578

Notes

*K*: average number of nucleotide differences; *S*: segregating sites; *θ*_W_ = Ne*µ* for the mtDNA locus and 4Ne*µ* for the nDNA locus, where Ne is the effective population size, and *µ* is the mutation rate per nucleotide (*θ*_Wnuc_) and per generation (*θ*_Wgen_); *π*: nucleotide diversity.

Significance: **0.01, *0.05, *^#^0.10*.

### 3.2 Step 1: Present and past geographic distributions

When constructing SDMs, a strong correlation was observed among some of the 19 bioclimatic variables (Supporting Information Figure S5.6). Three iterations of eliminating variables and factors with low contributions to the total variation were required until all retention criteria were met. Ultimately, four factors (MR1–4, *α* > 0.7; Supporting Information Figure S3.4) explained 100% of the variation in eight retained variables, and 84% of the variation in all 19 bioclimatic variables. Correlation among the four factors was lower than among the original variables in all four time periods considered (i.e., present, MH, LGM, and LIG; Supporting Information Table S5.6). For convenience, we named the four factors according to the original variables with which they were strongly correlated (*r* > 0.9; Supporting Information Figure S5.7; also see Supporting Information Figures S5.8 and S5.9).

**Figure 3:**
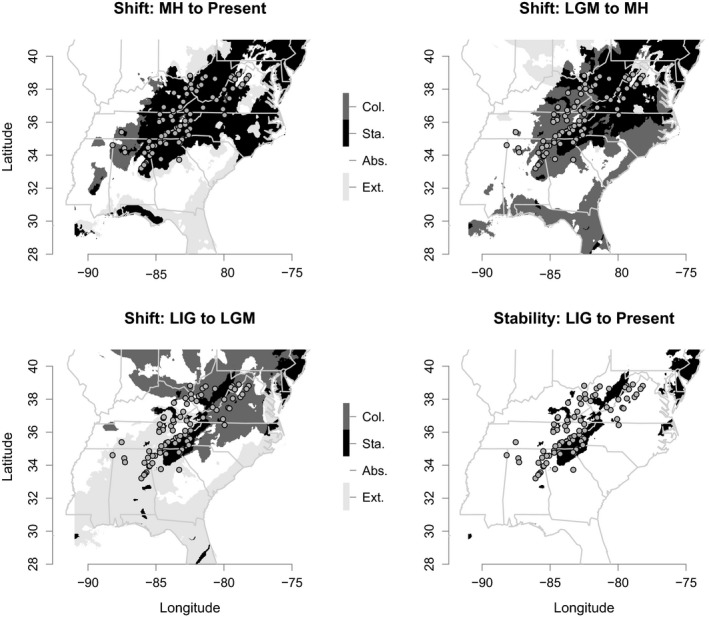
Distributional shifts and stability. Maps showing inferred distributional shifts and long‐term stability for successive time periods: MH to present, LGM to MH, and LIG to LGM. Each panel depicts four occurrence categories: colonization (Col.), stability (Sta.), absence (Abs.), and extinction (Ext.). The superimposed gray dots represent the 91 occurrence points used for distribution modeling

Distributional shift and stability maps (Figure 3) showed that: (a) from the LIG to the LGM, most of the suitable habitat shifted northward from the East Coast and the Gulf Coast toward the location of the southern edge of the Laurentide ice sheet, above 40° latitude; (b) from the LIG to the present, the southern edge of *R. flavipes*' distribution underwent a extinction‐colonization (or contraction‐expansion) cycle; (c) the eastern portion of West Virginia and areas around western North Carolina had suitable habitat from the LIG to the present; and (d) the amount of suitable habitat increased since the beginning of the Holocene.

### 3.3 Step 2: Genetic variation and the role of environment and space in genetic structuring

**Figure 4:**
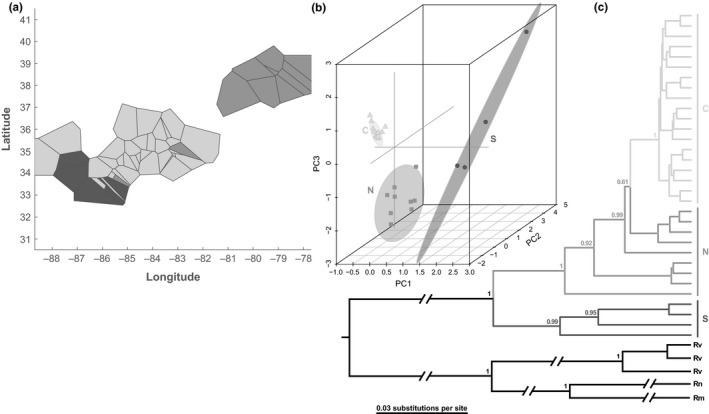
Identification of natural genetic populations based on mtDNA sequences. (a) Bayesian spatial‐genetic clustering. The map shows the inferred locations of three genetic clusters recovered using BAPS: Northern (gray), Central (light gray) and Southern (dark gray). (b) Principal Components Analysis. Principal component scores are shown in three dimensions with grouping of individuals according to the BAPS clusters. (c) *Bayesian Maximum Clade Credibility tree*. For the in‐group (*R. flavipes*), nodes and branches are shaded according to the BAPS clusters, and labels with abbreviations as follows: Northern (N), Central (C), and Southern (S). Only those node support values (posterior probabilities) >0.50 are shown. Abbreviations for out‐group taxa are: *R. virginicus* (Rv), *R. malletei* (Rm) and *R. nelsonae* (Rn)

The BAPS analysis identified three genetic clusters, each with largely separate geographic distributions (Figure 4a). Herein, we refer to them as the Northern, Central, and Southern clusters. We used the first three principal components (PCs) to represent these clusters in three dimensions (Figure 4b). The three PCs accounted for 53% of the variance at the mtDNA locus; they showed that the Northern cluster is most similar to the Central cluster. Phylogenetic reconstruction using BEAST produced a Bayesian tree (Figure 4c) that corroborated the three clusters identified using BAPS and PCA, albeit with the Northern cluster as paraphyletic. Molecular dating using the mtDNA locus in BEAST estimated the Southern‐Northern divergence at a median of 131.9 kya (95% CI: 83.6–195.0 kya; Supporting Information Figure S6.10), and the Northern‐Central divergence at a median of 35.8 kya (95% CI: 21.5–56.7 kya; Supporting Information Figure S6.10).

Although the Southern cluster comprised only four mtDNA haplotypes, this group had the most genetic variation (nucleotide diversity, *π* = 0.016; mean number of nucleotide differences, *K* = 17.50; Table 1). Nine mtDNA haplotypes in the Northern cluster resulted in values of *π* = 0.010, and *K* = 11.44, and, although there were 19 haplotypes in the Central cluster, these diversity values were lowest (i.e., *π* = 0.003 and *K* = 3.68; Table 1). Genetic differentiation was highest between Southern versus Central clusters (*F*_ST_ = 0.659) whereas Northern versus Central differentiation was lowest (Supporting Information Table S6.7).

Genetic structure was influenced by environment and geography. The full model of environmental and spatial structure predictors accounted for 58.7% of the observed genetic variation at the mtDNA locus. Spatial structure alone explained 41.1% (*p* < 0.001) of the genetic variation. Environmental factors accounted for 5.2% (*p* = 0.012) of the variation. The interaction between the two explained an additional 12.4% of the genetic variation. After removing the effect of spatial structure, the factors with significant contribution to genetic variation were “temperature range” and “wet‐season precipitation” (Supporting Information Figure S6.11).

### 3.4 Step 3: Phylogeographic hypothesis testing and population size changes

**Figure 5:**
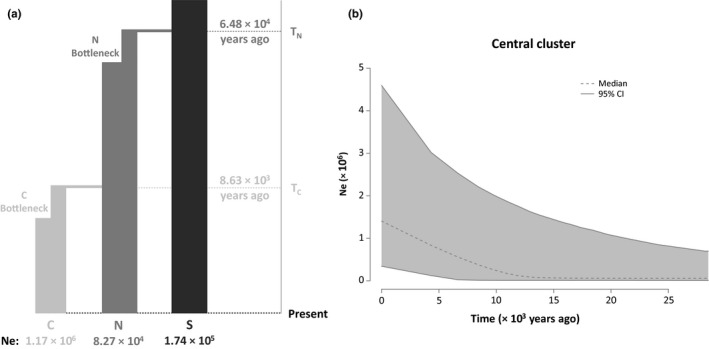
(a) Best‐fit phylogeographic scenario inferred using ABC. The distributional shift hypothesis represents a case where the Northern (N) cluster first diverged from the Southern (S) cluster, and the Central (C) cluster subsequently diverged from the Northern cluster, in a stepping‐stone fashion. Branch widths of the population tree represent effective population sizes (Ne), and the model includes brief bottlenecks associated with each founder event (see Section 2—Step 3). (b) Extended Bayesian skyline plot. The plot shows changes in effective population size (Ne) over time in the Central cluster, jointly estimated from mtDNA and nDNA data

In the two sets of first‐tier ABC comparisons: (a) the refuge‐based scenario with the highest posterior probability was the hypothesis that postulated the Northern region was the source from which the Southern cluster diverged first, followed by the Central cluster (scenario R3; Table 2; Supporting Information Figure S4.3); and (b) the distributional shift scenario that provided the best fit to the empirical data was the hypothesis that represented a case of Southern‐to‐Northern‐to‐Central stepping‐stone colonization (scenario DS1; Table 2; Supporting Information Figure S4.4). In the second tier of ABC comparisons, the best‐fit scenario was DS1 (Table 2; Supporting Information Figure S4.5). The DS1 scenario had a posterior probability of 0.932 when compared against other DS scenarios in the first tier, but its posterior probability in the second tier was 0.495 compared to 0.332 for the second‐best R3 scenario. Both of these scenarios had high type I and II error rates in the second‐tier comparisons (Supporting Information Table S7.8). Based on examination of estimated parameter values from the best‐fit model, divergence between the Northern and Southern populations was the oldest, estimated to have occurred 64.80 kya (95% CI: 26.40–115.00 kya; Figure 5a; Supporting Information Table S7.9), while the Northern and Central populations diverged 8.63 kya (95% CI: 2.75–22.50 kya; Figure 5a; Supporting Information Table S7.9).

**Table 2:** Two‐tiered ABC hypothesis testing

Refugial scenarios			Distributional shift scenarios			Refugium vs. Distributional shift vs. Vicariance		
Scenario	Posterior probability	95% CI	Scenario	Posterior probability	95% CI	Scenario	Posterior probability	95% CI
R1: S‐N;S‐C	0.103	0.087–0.120	DS1: S‐N;N‐C	**0.932**	**0.918–0.946**	R3: N‐S;N‐C	0.332	0.313–0.351
R2: S‐C;S‐N	0.014	0.010–0.018	DS2: S‐C;C‐N	0.002	0.001–0.003	DS1: S‐N;N‐C	**0.495**	**0.481–0.510**
R3: N‐S;N‐C	**0.861**	**0.843–0.879**	DS3: N‐S;S‐C	0.064	0.050–0.078	V: N/S;N‐C	0.173	0.159–0.187
R4: N‐C;N‐S	0.013	0.009–0.016	DS4: N‐C;C‐S	0.002	0.001–0.003			
R5: C‐S;C‐N	0.006	0.003–0.009	DS5: C‐S;S‐N	0.000	0.000–0.001			
R6: C‐N;C‐S	0.003	0.001–0.005	DS6: C‐N;N‐S	0.000	0.000–0.001			

Best‐fit scenarios are highlighted in bold font.

ABC hypothesis testing was performed in two tiers. In the first tier, refugial and distributional shift scenarios were evaluated separately. In the second tier, these two scenarios, as well as a vicariance scenario (V; Supporting Information Figure S5.9), were compared.

The Central population was the only cluster that showed a signature of population growth, based on significant results for Tajima's *D* (*D* = −1.90 for the mtDNA locus; Table 1), as well as Fu and Li's statistics (*D* = −2.24, *F* = −2.49; Table 1). Likewise, mismatch distribution analyses revealed evidence of population growth in the Central cluster only. This population experienced significant growth (*R* ^2^ = 0.047; *p* < 0.001), whereas no size changes were detected in the Northern (*R* ^2^ = 0.166; *p* = 0.479), or the Southern (*R* ^2^ = 0.154; *p* = 0.116) clusters. The EBSP assessments of changes in Ne over time also showed evidence of growth of the Central cluster, initiated in the last 10,000 years (Figure 5b). Furthermore, nonsignificant outcomes from compound neutrality tests for the mtDNA locus suggested that the aforementioned inferences were not obscured by selection (Supporting Information Table S8.10).

## 4 DISCUSSION

This study provides new insights into how Pleistocene climatic fluctuations impacted the geographic distribution of *R. flavipes* in the southern Appalachian Mountains and surrounding areas. The interplay between past climate change and complex montane topography, and its impact on the spatial distribution of intraspecific genetic diversity has been reported for other taxa from temperate regions (Hewitt, 2004). While there has been extensive work on salamanders from the southern Appalachians (Crespi, Rissler, & Browne, 2003; Jones & Weisrock, 2018; Jones, Voss, Ptacek, Weisrock, & Tonkyn, 2006; Kuchta, Haughey, Wynn, Jacobs, & Highton, 2016; Rissler & Smith, 2010; Zamudio & Savage, 2003), relatively few studies have focused on reconstructing the long‐term population history of forest‐dependent arthropods in this region (but see Hedin & Wood, 2002; Nalepa, Luykx, Klass, & Deitz, 2002; Thomas & Hedin, 2008; Walker, Stockman, Marek, & Bond, 2009; Caterino & Langton‐Myers, 2018). Indeed, the predominant focus on vertebrates and vascular plants in conservation research and planning is likely to result in management strategies that fail to cater to a large proportion of biodiversity (Garrick, Newton, & Worthington, 2018 and references therein). To understand drivers of phylogeographic patterns in *R. flavipes*, we examined evidence for distributional shifts using SDMs, and reconstructed the evolutionary and demographic history of *R. flavipes* using ABC analyses. Overall, we determined that the location of key refugia has changed over time (e.g., from one glacial period to the next), rather than a single refugium repeatedly serving as a reservoir of genetic diversity, whereby successive glacial‐interglacial cycles reinforce the same genetic signatures of contraction and expansion.

### 4.1 Climate change as a driver of distributional shifts and genetic divergence

Determining whether distributional shifts have occurred in the history of a species can lead to a better understanding of processes that have shaped present‐day genetic variation. Our SDMs suggested that in the period between the LIG and LGM, suitable habitat for *R. flavipes* shifted from the East Coast and the Gulf Coast northward toward the former southern edge of the Laurentide ice sheet (Figure 3). Consistent with this, our genetic analyses confirmed that the Northern cluster diverged between the LIG and LGM (ABC: 26.4–115.0 kya; BEAST: 83.6–195.0 kya). As suitable habitat expanded southward following the LGM (Figure 3), the Central cluster diverged during the LGM‐Holocene transition (ABC: 2.8–22.5 kya; BEAST: 21.5–56.7 kya) and continued to expand in the Holocene, both in terms of geographic range (Figure 3) and population size (Figure 5).

Our inferences about the long‐term population history of *R. flavipes* are not dissimilar from reconstructions of glacial‐interglacial colonization routes followed by many plant and animal species in the eastern U.S. For example, the pitcher‐plant mosquito, *Wyeomyia smithii*, initially dispersed from the Gulf Coast northward along the East Coast, and subsequently moved southward into the southern Appalachians (Merz et al., 2013). Similarly, the red salamander, *Pseudotriton ruber*, persisted in the Coastal Plain in the early Pliocene, and then expanded its range toward Appalachian upland habitat as cooling trends started in the early Pleistocene (Folt, Garrison, Guyer, Rodriguez, & Bond, 2016). Thus, despite different life history traits, at least a few forest‐dependent organisms may have responded similarly to climatic fluctuations in the past.

### 4.2 A northern refugium during the LGM and divergence of the Central cluster in the Holocene

Our analyses suggested that a northern refuge played a key role in subsequent colonization by *R. flavipes* of the central region of the southern Appalachians. Pollen records indicate that climatic conditions suitable for temperate forests existed over large areas of the southeastern U.S. during the LGM (Williams, Post, Cwynar, Lotter, & Levesque, 2002). Furthermore, fossil and genetic evidence suggest that some tree species, including red oak, red maple and beech, were widespread in this region during that time (Magni, Ducousso, Caron, Petit, & Kremer, 2005; McLachlan, Clark, & Manos, 2005). Although somewhat unexpected, the existence of northern refugia close to the southern edge of the Laurentide ice sheet during the LGM is plausible owing to localized warm areas in close proximity to glaciers (Bennett & Provan, 2008; Jackson et al., 2000; Magni et al., 2005; McLachlan et al., 2005; Rowe, Heske, Brown, & Paige, 2004; Williams et al., 2002).

Despite the broad geographic range of the *R. flavipes* Central cluster (Figure 4a), this group contained the lowest genetic diversity (Table 1). We suggest that this is likely the result of founder effects associated with the relatively recent colonization of the central portion of the southern Appalachians from the north. Although subsequent population expansion seems to have occurred in the central region, more time may be needed to replace lost genetic variation. Assessment of changes in Ne over time showed that the Central cluster had increased in size over the last 10,000 years (Figure 5b), which is consistent with inferences based on nongenetic data that indicated the amount of suitable habitat in the central region increased since the LGM (Figure 3).

### 4.3 The potential role of environmental variables in promoting range expansions

Given the desiccation susceptibility of soft‐bodied arthropods, range expansions and population growth in *R. flavipes* may be have been influenced by local‐scale site‐specific environmental variables such as precipitation. The southeastern U.S. was much warmer during the mid‐Holocene (cf. LGM; Bartlein et al., 1998), when tupelo and oak forest types dominated over pine, indicating wetter conditions (LaMoreaux, Brook, & Knox, 2009). The *R. flavipes* Central cluster likely diverged from the Northern cluster following a cooling trend in the Younger Dryas (~12.9–11.7 kya). While this was a global cooling period, locally in the southeastern U.S., this period was characterized by a warmer and wetter climate, reflecting the trapping of heat in the western subtropical gyre due to reduced Atlantic meridional overturning circulation (Grimm et al., 2006). Accordingly, if high precipitation was important for facilitating range expansion, these conditions seem to have been in place at a time that coincides with colonization of the central region. Furthermore, seasonal differences in precipitation between the southern and northern portions of the study region (Hyseni & Garrick, 2019) may have led to different flight phenologies and thus seasonal isolation and niche partitioning. Consistent with this, dbRDA revealed that in addition to spatial structuring of genetic variation, wet‐season precipitation accounted for the remainder of genetic differentiation of the Southern cluster compared to the other two. We suggest that the influence of local‐scale environmental variables upon the capacity for termite population growth and range expansion warrants further investigation.

### 4.4 The influence of spatial scale on genetic structure

Compared to previous work on *R. flavipes*, the spatial scale over which we detected genetic structure is notable. For example, based on mtDNA sequence and microsatellite genotypic data, Perdereau et al. (2013) identified three distinct genetic clusters of *R. flavipes* in the eastern and southeastern U.S. across an area spanning at least twice the distance covered by sampling in the present study. However, with the exception of a few collection sites in West Virginia, those authors did not include *R. flavipes* sampled from the southern Appalachians. This contrast supports the view that fine‐scale genetic structuring may be particularly prevalent in topographically complex montane areas (Garrick, 2011; Hedin & Wood, 2002; Thomas & Hedin, 2008). Along a ~1,000 km transect traversing the southern Appalachians, a wood‐feeding cockroach (*Cryptocercus punctulatus*) that is syntopic with *R. flavipes* consists of five distinct genetic groups (Everaerts et al., 2008; Garrick, Sabree, Jahnes, & Oliver, 2017). Interestingly, both of these saproxylic taxa have a zone of parapatry between genetic groups in the central region. Comparative phylogeographic analyses would be informative about the extent to which spatial‐genetic patterns seen in dead‐wood‐associated insects correspond with shared microevolutionary processes that underpin them.

### 4.5 Caveats and future directions

An early understanding of genetic consequences of Pleistocene range expansions came from study systems that either repeatedly experienced severe glaciation (Hewitt, 1996), or were relatively simplified linear systems (Nason, Hamrick, & Fleming, 2002). In these cases, unidirectional expansion out of a single major refuge was commonly inferred, often based on signatures of repeated founder effects and serial reduction in genetic diversity at the leading edge. However, an expanded view of the geography of range expansion may be needed when considering unglaciated, topographically complex, montane landscape settings. In this study, we considered distributional shift (see Section 1) to be a plausible phylogeographic scenario for the southern Appalachian Mountains. However, further work is needed to understand the circumstances under which distributional shift scenarios are distinguishable from single‐refuge contraction‐expansion scenarios. Indeed, inferring Pleistocene distributional shifts using genetic data can be challenging, as multiple historical factors can contribute to current genetic variation.

Although our ABC analyses identified distributional shift as the best‐fit scenario, it did not receive unambiguously superior support relative to the next‐best scenario, and the estimated error in scenario choice was large (Supporting Information Table S7.8). Accordingly, we must consider our ABC‐based inference to be a preliminary working hypothesis, to be re‐evaluated and re‐tested with new data. Notwithstanding some limitations of our ABC inferences, it is notable that a common feature of the best‐fit and second‐best hypotheses is the expansion of the Central cluster. Specifically, both scenarios include the Northern cluster giving rise to the Central cluster. Additionally, both scenarios include a direct long‐distance dispersal event. Buckley (2009) advocated for an iterative approach to phylogeography, highlighting the value of working hypotheses for focusing subsequent analytical efforts on scenarios that have some empirical support. This study contributes to a growing body of literature that highlights an important role for multiple refugia—including those located further north than previously expected—in phylogeographic structuring of plants (McLachlan et al., 2005), vertebrates (Fontanella, Feldman, Siddall, & Burbrink, 2008), and invertebrates (Merz et al., 2013). Having characterized contemporary fine‐scale spatial structure and historical climate‐based distributions for *R. flavipes*, the present study has also revealed specific geographic locations that warrant dedicated sampling (e.g., the Southern genetic cluster has a relatively small range that requires better representation, and based on SDMs, sampling in the Gulf Coast and Coastal Plain areas would be particularly valuable).

## CONFLICT OF INTEREST

None declared.

## AUTHOR CONTRIBUTIONS

Both authors conceived the study, and collected and curated samples from across the southern Appalachians; C.H. collected DNA sequence data, performed the analyses, and wrote the first draft; both authors edited subsequent drafts.

## Data Availability

All appendices are included in Supporting Information—File S1 (Supplementary Methods and Supplementary Results). All DNA sequence data are included in Supporting Information—File S2, with Genbank accession numbers provided in the file. Posterior probabilities and error rates for all phylogeographic hypotheses tested in this study are included in Supporting Information—File S3. The Supporting Information and additional SDM, BAPS, BEAST, and ABC data are available for download from DRYAD via http://datadryad.org under repository entry https://doi.org/10.5061/dryad.5hr7f31.
