# Supplementary material for: The role of glacial‐interglacial climate change in shaping the genetic structure of eastern subterranean termites in the southern Appalachian Mountains, USA
Source: Ecol Evol. 2019 Apr 1;9(8):4621–36. doi: 10.1002/ece3.5065 (PMC6476779; doi:10.1002/ece3.5065)
Supplement: Supplementary file 1 [file ECE3-9-4621-s001.docx]

**Supplementary Methods**

**Appendix S1.** Population sampling.

*Reticulitermes flavipes* termites were collected for genetic analyses between 2012 and 2014 from 46 locations in the southern Appalachian Mountains. The presence of *R. flavipes* was confirmed at an additional 45 locations from 2015 to 2016. Since it is not possible to reliably distinguish among several co-distributed species on the basis of morphology when only members of the worker caste are collected (Wang et al. 2009), termites were identified using a molecular assay (Garrick et al. 2015). Briefly, a short (376-bp) region of the mitochondrial COII gene was amplified (using PCR primers RetCo2-F and RetCo2-R), and products were then separately digested with three restriction enzymes (RsaI, TaqI, and MspI), which in combination generate diagnostic species-specific banding patterns.

**Table S1.1.** *Geographic locations from which* Reticulitermes flavipes *termites were sampled.* Each site has a unique ID, and associated state and county information is shown. Spatial coordinates are reported in decimal degrees, and elevation is in meters. Occurrence of *R. flavipes* was confirmed at 91 sites, and these were all used for Species Distribution Modeling. Genetic data were collected from individuals sampled from the first 46 sites.

| **Site ID** | **State** | **County** | **Longitude** | **Latitude** | **Elevation** | **Genetic Data** |
| --- | --- | --- | --- | --- | --- | --- |
| A03 | Georgia | Gilmer | -84.63805 | 34.77972 | 764 | Yes |
| A04 | Georgia | Gordon | -85.06536 | 34.57297 | 450 | Yes |
| A09 | Georgia | Chattooga | -85.24268 | 34.56515 | 408 | Yes |
| A13 | Alabama | Cleburne | -85.70074 | 33.56059 | 425 | Yes |
| A14 | Alabama | Clay | -85.81731 | 33.46215 | 485 | Yes |
| A16 | Alabama | Clay | -86.07201 | 33.2015 | 291 | Yes |
| A18 | Georgia | Murray | -84.71137 | 34.87866 | 354 | Yes |
| A21 | Georgia | Gilmer | -84.3388 | 34.77507 | 730 | Yes |
| A22 | Georgia | Fannin | -84.25093 | 34.68311 | 810 | Yes |
| A30 | Tennessee | Sevier | -83.51849 | 35.65682 | 780 | Yes |
| A31 | Tennessee | Sevier | -83.35717 | 35.70232 | 653 | Yes |
| A32 | North Carolina | Swain | -83.31077 | 35.52117 | 666 | Yes |
| A37 | Tennessee | Cocke | -83.21343 | 35.7714 | 575 | Yes |
| A40 | Georgia | Murray | -84.6914 | 34.75931 | 804 | Yes |
| A41 | Alabama | Cleburne | -85.49764 | 33.91858 | 257 | Yes |
| A52 | Virginia | Giles | -80.54509 | 37.34757 | 1121 | Yes |
| A56 | Virginia | Albemarle | -78.78368 | 38.12902 | 814 | Yes |
| A60 | Virginia | Greene | -78.64308 | 38.29123 | 761 | Yes |
| A62 | Virginia | Rappahannock | -78.18149 | 38.80508 | 755 | Yes |
| A64 | Virginia | Madison | -78.3406 | 38.62592 | 1032 | Yes |
| A70 | Virginia | Augusta | -79.3498 | 38.04052 | 784 | Yes |
| A73 | Tennessee | Lawrence | -87.52677 | 35.39384 | 304 | Yes |
| A75 | Tennessee | Morgan | -84.74478 | 36.12452 | 378 | Yes |
| A76 | Tennessee | Morgan | -84.48829 | 36.13606 | 496 | Yes |
| A85 | Georgia | Dade | -85.49971 | 34.84695 | 315 | Yes |
| A86 | Mississippi | Tishomingo | -88.19299 | 34.60502 | 177 | Yes |
| A87 | Tennessee | Monroe | -84.2476 | 35.34883 | 327 | Yes |
| A88 | Tennessee | Monroe | -84.19383 | 35.34534 | 425 | Yes |
| A92 | North Carolina | Swain | -83.59187 | 35.32969 | 593 | Yes |
| A97 | North Carolina | Buncombe | -82.48742 | 35.59535 | 722 | Yes |
| A106 | West Virginia | Pendleton | -79.38618 | 38.82374 | 528 | Yes |
| A107 | West Virginia | Pendleton | -79.38506 | 38.82585 | 548 | Yes |
| A108 | West Virginia | Pendleton | -79.48494 | 38.72694 | 936 | Yes |
| A117 | Alabama | Clay | -85.80658 | 33.47105 | 621 | Yes |
| A124 | Alabama | Clay | -85.87318 | 33.40451 | 460 | Yes |
| A131 | Alabama | Lawrence | -87.33273 | 34.41979 | 321 | Yes |
| A133 | Alabama | Winston | -87.2768 | 34.17659 | 248 | Yes |
| A134 | Alabama | DeKalb | -85.58357 | 34.4554 | 395 | Yes |
| A137 | Alabama | Cherokee | -85.4573 | 33.9634 | 300 | Yes |
| A138 | Alabama | Etowah | -85.84679 | 34.14676 | 188 | Yes |
| A139 | Georgia | Floyd | -85.26428 | 34.1226 | 232 | Yes |
| A141 | South Carolina | Oconee | -83.10755 | 34.862 | 536 | Yes |
| A142 | South Carolina | Jackson | -83.05563 | 35.01376 | 887 | Yes |
| A143 | South Carolina | Oconee | -83.08929 | 34.94523 | 744 | Yes |
| A145 | South Carolina | Oconee | -83.22783 | 34.72782 | 394 | Yes |
| A146 | South Carolina | Oconee | -83.31242 | 34.77755 | 469 | Yes |
| A150 | Georgia | Greene | -83.29258 | 33.72088 | 132 | No |
| T1 | Virginia | Scott | -82.74536 | 36.70494 | 460 | No |
| T2 | Virginia | Botetourt | -79.68214 | 37.47978 | 759 | No |
| T3 | Virginia | Smyth | -81.53171 | 36.88458 | 731 | No |
| T4 | Virginia | Patrick | -80.06615 | 36.78941 | 386 | No |
| T6 | North Carolina | Rockingham | -79.9509 | 36.43191 | 256 | No |
| T10 | Virginia | Bedford | -79.59341 | 37.4409 | 692 | No |
| T12 | Virginia | Bath | -79.97707 | 37.98723 | 508 | No |
| T13 | West Virginia | Pendleton | -79.2019 | 38.60225 | 591 | No |
| T15 | West Virginia | Hardy | -78.91022 | 38.89373 | 589 | No |
| T16 | Ohio | Gallia | -82.49056 | 38.81387 | 277 | No |
| T17 | Tennessee | Morgan | -84.75872 | 36.01773 | 572 | No |
| T19 | Tennessee | Scott | -84.7143 | 36.47398 | 479 | No |
| T20 | Kentucky | McCreary | -84.45749 | 36.84983 | 412 | No |
| T21 | Kentucky | McCreary | -84.4248 | 36.91024 | 336 | No |
| T22 | Tennessee | Knox | -83.76402 | 36.10415 | 399 | No |
| T23 | Tennessee | Union | -83.89043 | 36.37519 | 490 | No |
| T24 | Kentucky | Bell | -83.69725 | 36.60349 | 352 | No |
| T25 | Kentucky | Bell | -83.74413 | 36.72807 | 390 | No |
| T26 | Kentucky | Harlan | -83.21425 | 36.92808 | 767 | No |
| T27 | Tennessee | Sullivan | -82.48692 | 36.49101 | 427 | No |
| T29 | Kentucky | Knott | -82.99386 | 37.24096 | 318 | No |
| T31 | Georgia | Douglas | -84.63633 | 33.76154 | 295 | No |
| T32 | North Carolina | Buncombe | -82.49127 | 35.60575 | 770 | No |
| T33 | North Carolina | Henderson | -82.71758 | 35.44758 | 1205 | No |
| T34 | North Carolina | Henderson | -82.58961 | 35.21877 | 809 | No |
| T35 | Tennessee | Monroe | -84.24123 | 35.34314 | 413 | No |
| T36 | Tennessee | Monroe | -84.11201 | 35.39665 | 553 | No |
| T37 | Tennessee | Polk | -84.33586 | 35.20793 | 513 | No |
| T39 | Tennessee | Polk | -84.60815 | 35.14822 | 588 | No |
| T46 | North Carolina | Madison | -82.84724 | 35.85284 | 656 | No |
| T47b | Tennessee | Greene | -82.84973 | 36.08371 | 408 | No |
| T48 | Tennessee | Unicoi | -82.44664 | 36.10384 | 522 | No |
| T55 | Kentucky | Powell | -83.67732 | 37.77913 | 256 | No |
| T57 | Kentucky | Floyd | -82.72829 | 37.71582 | 213 | No |
| T58 | Kentucky | Lawrence | -82.82529 | 38.05997 | 209 | No |
| T59 | West Virginia | Wayne | -82.42619 | 38.30313 | 186 | No |
| T60 | West Virginia | Wayne | -82.38316 | 38.02512 | 402 | No |
| T61 | West Virginia | Logan | -82.01469 | 37.88885 | 260 | No |
| T62 | West Virginia | Lincoln | -81.84275 | 38.18754 | 201 | No |
| T63 | West Virginia | Kanawha | -81.66953 | 38.26121 | 267 | No |
| T64 | West Virginia | Jackson | -81.57557 | 38.652 | 241 | No |
| T65 | West Virginia | Roane | -81.3447 | 38.77533 | 240 | No |
| T66 | West Virginia | Braxton | -80.65887 | 38.63269 | 394 | No |
| T68 | West Virginia | Summers | -80.83122 | 37.50894 | 550 | No |

**Table S1.2.** *Geographic locations from which* Reticulitermes *out-group taxa were sampled.* Site ID and associated state and county information is shown Spatial coordinates are reported in decimal degrees, and elevation is in meters.

| **Site ID** | **State** | **County** | **Longitude** | **Latitude** | **Elevation** | **Species** |
| --- | --- | --- | --- | --- | --- | --- |
| A06 | Georgia | Walker | -85.2163 | 34.64336 | 386 | *R. malletei* |
| A12 | Alabama | Cleburne | -85.69391 | 33.57157 | 328 | *R. nelsonae* |
| A10 | Georgia | Chattooga | -85.24043 | 34.56416 | 427 | *R. virginicus* |
| A146 | South Carolina | Oconee | -83.31242 | 34.77755 | 469 | *R. virginicus* |
| A25 | Georgia | White | -83.73265 | 34.74192 | 766 | *R. virginicus* |

**Appendix S2.** DNA isolation and genetic markers.

Mitochondrial cytochrome c oxidase subunit I (COI) and II (COII) genes, and an intronic portion of the nuclear endo-beta-1,4-glucanase (EB14G) gene, were targeted. Each of these DNA regions were amplified separately via Polymerase Chain Reaction (PCR) in 15 µL volumes containing 5 to 50 ng of genomic DNA, 5 picomoles of each of two primers (Table S2.3), and the following amounts of Promega (Madison, WI) reagents: 0.8 nanomoles of each dNTP, 32 nanomoles of MgCl_2_, 0.5 units of Go*Taq*, and 5 µg of bovine serum albumin, in a 1x final concentration of PCR buffer. Reactions were performed in a Bio-Rad (Hercules, CA) T100 Thermal Cycler with the following conditions: initial denaturation at 95 °C for 3 min, 35 cycles of 95 °C for 30 s, 52 °C for 30 s, and 72 °C for 1 min, followed by a final extension at 72 °C for 5 mins. PCR products were viewed following agarose gel electrophoresis and cleaned with ExoSAP-IT (USB, Cleveland, OH).

**Table S2.3.** *Primer sequences and locus information.* Primer sequences and their sources are reported here, including length of quality-filtered, trimmed mitochondrial (mtDNA) and nuclear (nDNA) sequence alignments measured in base pairs (bp).

|  | Gene region | Primers | | | Alignment Length |
| --- | --- | --- | --- | --- | --- |
|  |  | Name | Sequence | Source |  |
| mtDNA | COI | LCO-1490 | 5’- GGTCAACAAATCATAAAGATATTGG-3’ | Folmer *et al.*, 1994 | 563 bp |
|  |  | HCO-2198 | 5’- TAAACTTCAGGGTGACCAAAAAATCA -3’ | Folmer *et al.*, 1994 |  |
|  | COII | CO2-forward | 5’- AGAGCWTCACCTATTATAGAAC-3’ | Park *et al.*, 2004 | 554 bp |
|  |  | TK-N-3785 | 5’- GTTTAAGAGACCAGTACTTG -3’ | Simon *et al.*, 1994 |  |
| nDNA | EB14G | Ret_EB14G_F | 5'-ATGGAGGTCGCAGCTACGTC-3' | This study | 251 bp |
|  |  | Ret_EB14G_R | 5'-GGCGCTGTTGTACGTGTTCCAG-3' | This study |  |

**Appendix S3.** Construction of species distribution models.

*Model evaluation and calibration.* We used the ‘biomod2’ package (Thuiller et al. 2009) in R for Species Distribution Model (SDM) construction. We used presence records, and pseudo-absence points selected following Barbet-Massin et al. (2012), who showed that for machine learning methods it is better to use multiple replicates of pseudo-absence points, with the number of pseudo-absences in each replicate close to the number of occurrence points. Thus, we first ran a rectilinear surface range envelope model (Thuiller et al. 2009), and then, from outside the area predicted as suitable habitat, we picked 100 random points, creating 20 independent sets of pseudo-absences, each of which were combined with the same 91 presence records. Four modeling algorithms were run: artificial neural networks (Ripley 1996), generalized boosted models or boosted regression trees (Friedman 2001), random forest (Breiman 2001), and maximum entropy (Phillips et al. 2006). We used 5 cross-validation runs per algorithm, for a total of 400 runs (4 algorithms x 5 cross-validations x 20 datasets), with 5,000 iterations per run. To assess model performance, 75% of the data were used for training, with 25% set aside as "out-of-bag" test data. To maximize the accuracy of presence/absence classification, we used the True Skill Statistic (TSS = sum of sensitivity and specificity – 1; Allouche et al. 2006), where SDMs with mean TSS above 0.2 were retained. We then used the ensemble framework (Buisson et al. 2010) to obtain a weighted average of all SDMs, where SDMs were weighted according to TSS values.

*Climate data.* Present-day SDMs were based on mean climatological data spanning a period from 1960–1990, with all variables used at 1-km resolution. Historical distributions were modeled for the Mid-Holocene (MH; ~6 thousand years ago, kya), the Last Glacial Maximum (LGM, ~22 kya), and the Last Interglacial (LIG, 120–140 kya). For each period, 19 bioclimatic variables (Hijmans et al. 2005) were obtained from the WorldClim database v.1.4 (http://www.worldclim.org). Using the 1960–1990 climatological data as the baseline, MH and LGM paleoclimatic data were downscaled from simulations with Global Climate Models, from CMIP5 (http://cmip-pcmdi.llnl.gov/cmip5). LIG paleoclimatic data were downscaled from Otto-Bliesner et al. (2008).

*Factor analysis.* To reduce the number of predictors, and correlation among them, we performed factor analysis in successive stages using the ‘psych’ package (Revelle 2018), until two criteria were met: 1) each factor must be highly correlated (absolute value of r > 0.5) with at least two variables, and 2) each variable must be highly correlated with only one factor and show low correlation (absolute value of r < 0.3) with any other factor. We used ordinary least squares to find the minimum residual (MR) solution (Harman and Jones 1966). Oblique rotations were used, since strong correlations between factors were expected. Cattell’s (1966) scree test and Horn’s (1965) parallel analysis determined the number of factors to retain, and these were then inspected for reliability using Cronbach’s (1951) α, with an acceptance criterion of α > 0.7.

*Factor names.* MR1: “Temperature Range” (TR; strongly correlated with bio4: “Temperature Seasonality” and bio7: “Temperature Annual Range”); MR2: “Dry-season Precipitation” (DP; strongly correlated with bio14: “Precipitation of Driest Month” and bio17: “Precipitation of Driest Quarter”); MR3: “Summer Temperature” (ST; strongly correlated with bio5: “Maximum Temperature of Warmest Month” and bio10: “Mean Temperature of Warmest Quarter”); MR4: “Wet-season Precipitation” (WP; strongly correlated with bio13: “Precipitation of Wettest Month” and bio17: “Precipitation of Wettest Quarter”).

**Table S3.4.** *Environmental data by category (precipitation and temperature)*. The bioclimatic variables shown here represent four different periods: present-day, Mid-Holocene, Last Glacial Maximum, and Last Interglacial. All data were obtained from WorldClim 1.4. All environmental variables have been scaled to 1-km resolution.

| Category | Environmental Variable | Code | Abbreviation |
| --- | --- | --- | --- |
| Precipitation | Annual Precipitation | BioClim 12 | bio12 |
|  | Precipitation of Wettest Month | BioClim 13 | bio13 |
|  | Precipitation of Driest Month | BioClim 14 | bio14 |
|  | Precipitation Seasonality | BioClim 15 | bio15 |
|  | Precipitation of Wettest Quarter | BioClim 16 | bio16 |
|  | Precipitation of Driest Quarter | BioClim 17 | bio17 |
|  | Precipitation of Warmest Quarter | BioClim 18 | bio18 |
|  | Precipitation of Coldest Quarter | BioClim 19 | bio19 |
| Temperature | Annual Mean Temperature | BioClim 1 | bio1 |
|  | Mean Diurnal Range | BioClim 2 | bio2 |
|  | Isothermality | BioClim 3 | bio3 |
|  | Temperature Seasonality | BioClim 4 | bio4 |
|  | Max. Temperature of Warmest Month | BioClim 5 | bio5 |
|  | Min Temperature of Coldest Month | BioClim 6 | bio6 |
|  | Temperature Annual Range | BioClim 7 | bio7 |
|  | Mean Temperature of Wettest Quarter | BioClim 8 | bio8 |
|  | Mean Temperature of Driest Quarter | BioClim 9 | bio9 |
|  | Mean Temperature of Warmest Quarter | BioClim 10 | bio10 |
|  | Mean Temperature of Coldest Quarter | BioClim 11 | bio11 |


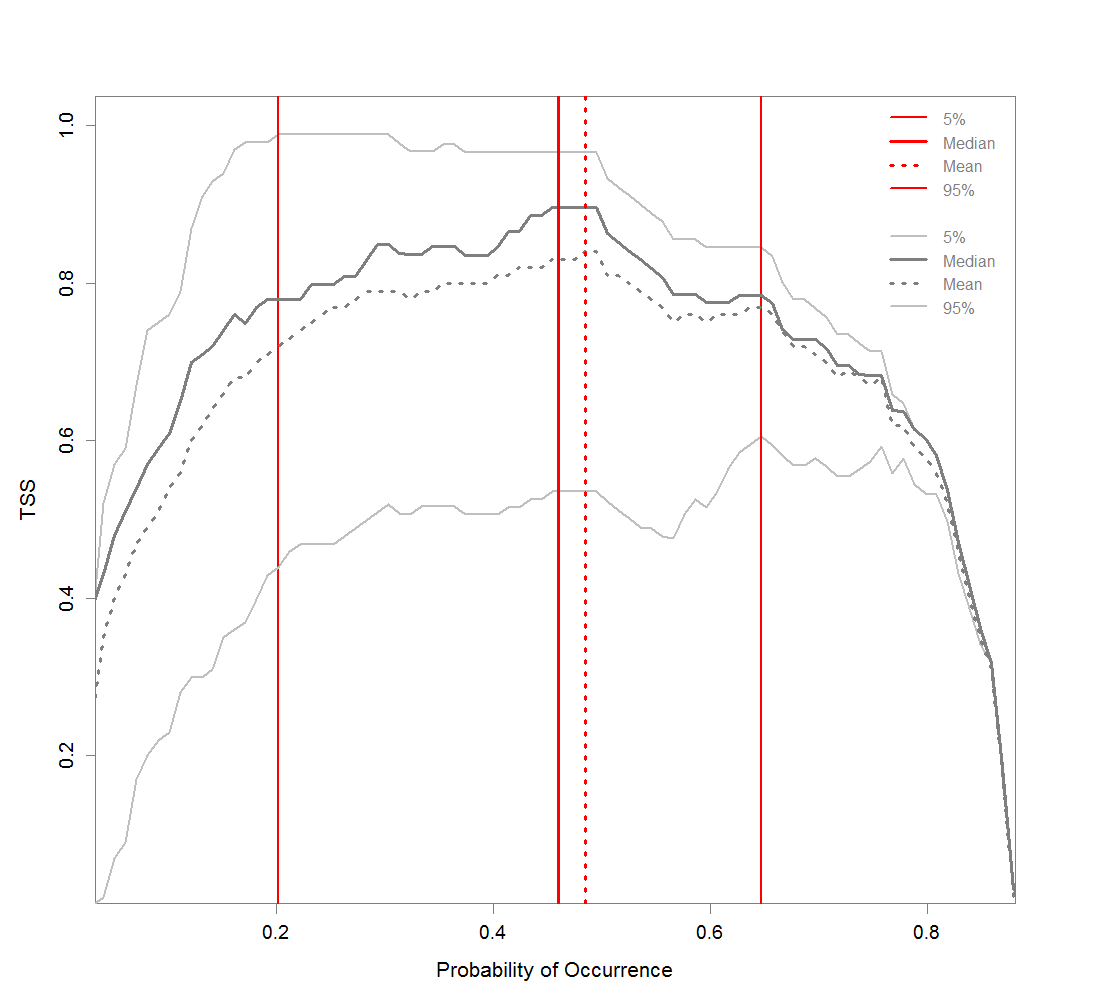


**Figure S3.1.** *Optimal probability of occurrence threshold for conversion to binary presence/absence.* For each probability of occurrence value, True Skill Statistic (TSS; equal to the sum of sensitivity and specificity – 1) was calculated based on 91 occurrence records and 100 pseudo-absence points. We computed confidence intervals using 20 pseudo-absence replicates.


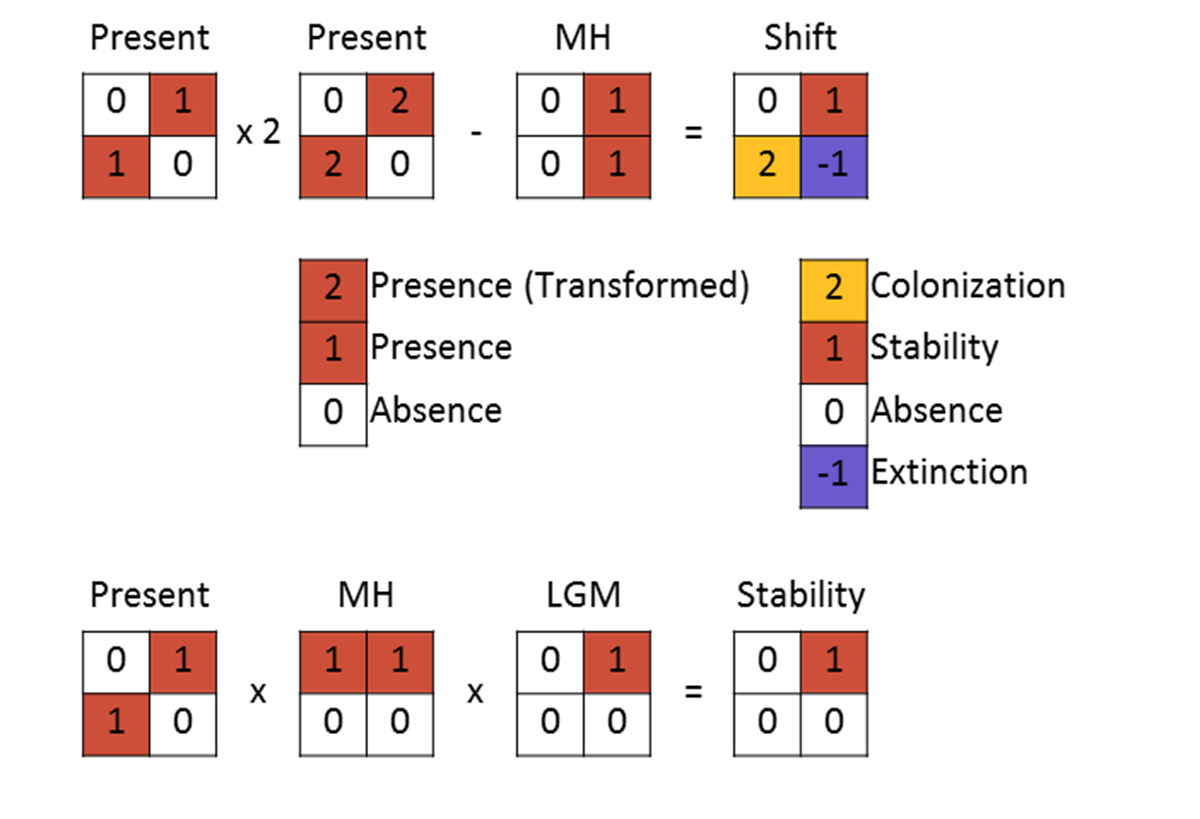


**Figure S3.2.** *Schematic of distributional shift and stability calculations.* Occurrence probability was converted to binary occurrence (0 = absence; 1 = presence) based on a threshold of 0.2. To calculate the distributional shift from the Mid-Holocene (MH) to the present, we took the difference of the two, after multiplying the binary occurrence map for the present by 2. This multiplication ensures that we obtain four categories in the distributional shift calculation: colonization (difference = 2), stability (1), absence (0), and extinction (-1). To calculate stability across several time periods, we multiplied the binary occurrence maps. The Last Glacial Maximum is abbreviated as LGM.

**Appendix S4.** Comparison of scenarios using approximate Bayesian computation

To identify the best-fit model, we used approximate Bayesian computation (ABC; Beaumont et al. 2002), implemented in DIYABC v.2.1.0 (Cornuet et al. 2014). Within the ABC framework, two classes of model parameters were used to characterize the phylogeographic hypotheses described above: effective population sizes (Ne), and divergence times (T). We performed two rounds of modeling: 1) the preliminary round with broad priors, and 2) the final round with narrower priors. Based on posterior probabilities from the preliminary round, for the Northern and Southern clusters, we used uniform priors of Ne = 25,000–250,000. For the Central cluster, posterior probabilities from the preliminary round were not informative for narrowing Ne range, so we used a broad log-uniform prior of Ne = 500,000–5,000,000. All competing scenarios had two divergence events: any two of T_N_, T_C_ or T_S,_ (the subscript is the first letter abbreviation of the new cluster, i.e., Northern, Central, or Southern). The prior range for the more recent event encompassed the Mid-Holocene (MH) and the Last Glacial Maximum (LGM) (i.e., T = 2,000–25,000 years ago), while the priors of the older event ranged from the LGM to the Last Interglacial (LIG) (i.e., T = 20,000–120,000). Given the overlap between these divergence time priors, we enforced a condition such that the latter event was required to occur before the former. *Reticulitermes* *flavipes* colonies produce alates once a year, approximately two years after colony foundation (Feytaud 1920), but colonies can grow to 70 individuals in their first year (Beard 1974). Thus, we assumed a 1-year generation time. We included brief bottlenecks (1–10 generations duration) at the beginning of each divergence event, in order to mimic founder events.

**Table S4.5***. ABC priors.* N, C, and S represent the effective population sizes of the Northern, Central, and Southern clusters. T_N,_ T_C,_ and T_S_ represent the time of divergence of N, C, and S. The parameters b_N,_ b_C,_ and b_S_ represent duration (number of generations) of bottleneck events, whereas N_b,_ C_b,_ and S_b_ represent effective population sizes during bottleneck events. In the vicariance scenario (see Fig. S5.10), N_Anc_ and T_SN_ are the effective population size before divergence, and time of divergence of the ancestor of S and N. The parameters µ_mt_ and µ_nuc_ are mutation rates of the mtDNA and nDNA loci.

| ***Parameter*** | ***Distribution*** | ***Minimum*** | ***Maximum*** |
| --- | --- | --- | --- |
| N | Uniform | 25,000 | 250,000 |
| C | Log-Uniform | 500,000 | 5,000,000 |
| S | Uniform | 25,000 | 250,000 |
| T_N_ or T_S_ or T_SN_ | Uniform | 20,000 | 120,000 |
| T_C_ | Uniform | 2,000 | 25,000 |
| b_N_ | Uniform | 1 | 10 |
| b_C_ | Uniform | 1 | 10 |
| b_S_ | Uniform | 1 | 10 |
| N_b_ | Log-Uniform | 500 | 50,000 |
| C_b_ | Log-Uniform | 100 | 10,000 |
| S_b_ | Log-Uniform | 500 | 50,000 |
| N_Anc_ | Log-Uniform | 5,000 | 500,000 |
| µ_mt_ | Uniform | 5 x 10^-9^ | 5 x 10^-7^ |
| µ_nuc_ | Uniform | 5 x 10^-10^ | 2.5 x 10^-8^ |
|  |  |  |  |
|  |  |  |  |
|  |  |  |  |
|  |  |  |  |


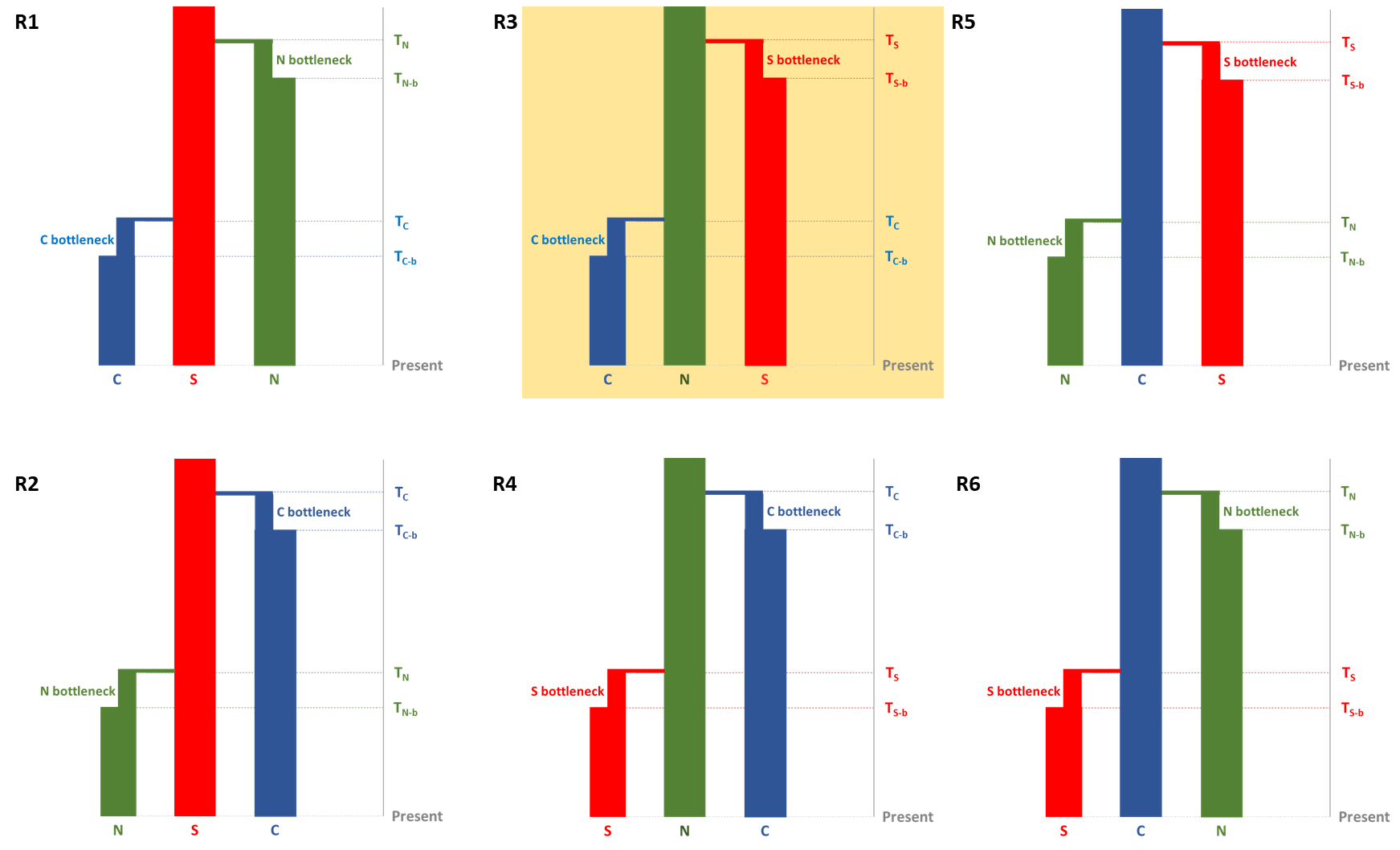


**Figure S4.3.** *Refugial scenarios.* Scenarios compared in the first step of the first tier of ABC analyses. These “refugial scenarios” involved persistence in a single refugium, such that the other areas were colonized via successive expansions out of that refugium. We considered three refugial locations: Southern (S) = red, Northern (N) = green, and Central (C) = blue.


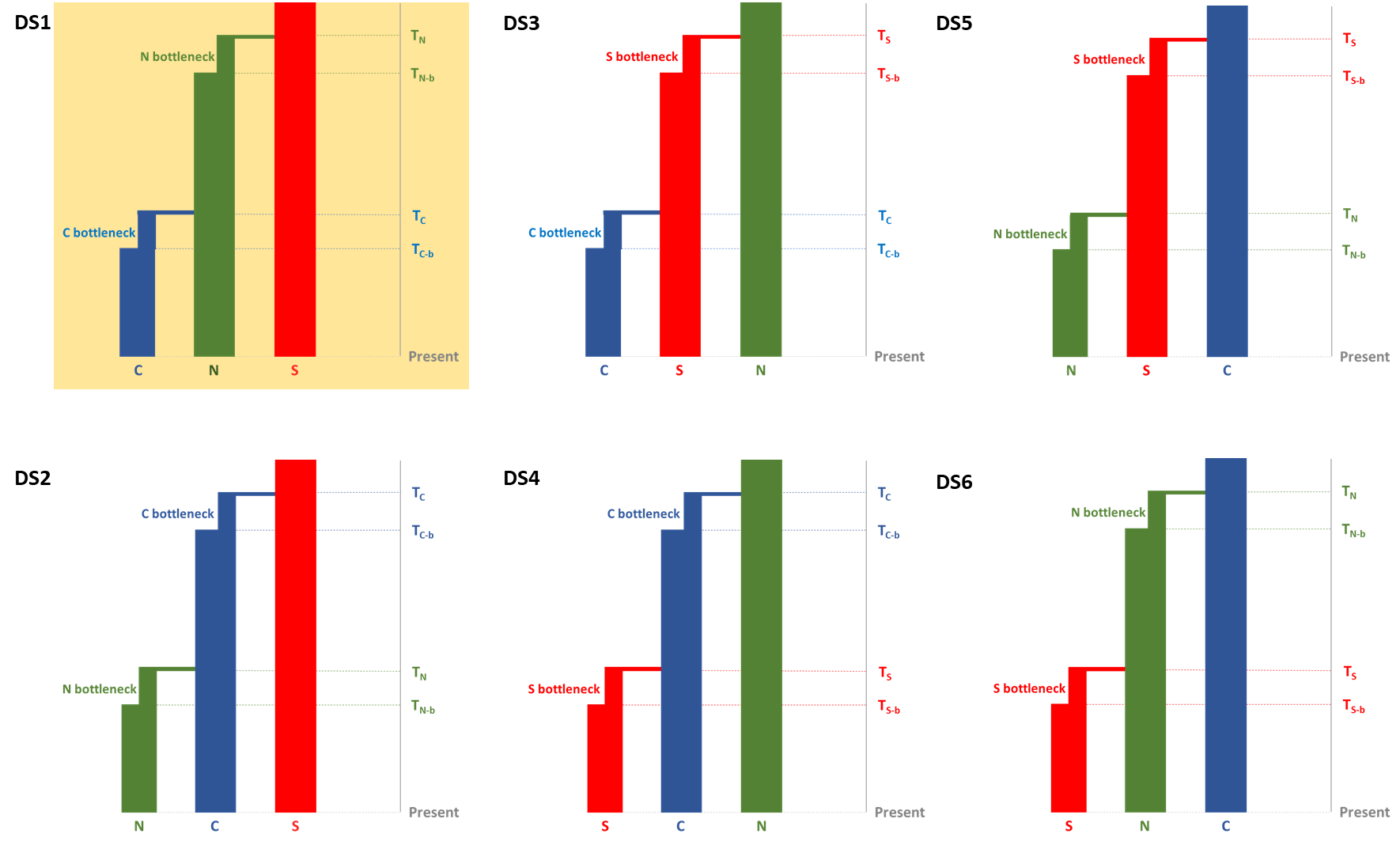


**Figure S4.4.** *Distributional shift scenarios.* Scenarios compared in the second step of the first tier of ABC analyses. “Distributional shift” scenarios involved divergence in a stepping-stone fashion, where one population gave rise to a descendant population, which later became the progenitor of the third population. The Southern (S) cluster is shown in red, the Northern (N) in green, and the Central (C) in blue.


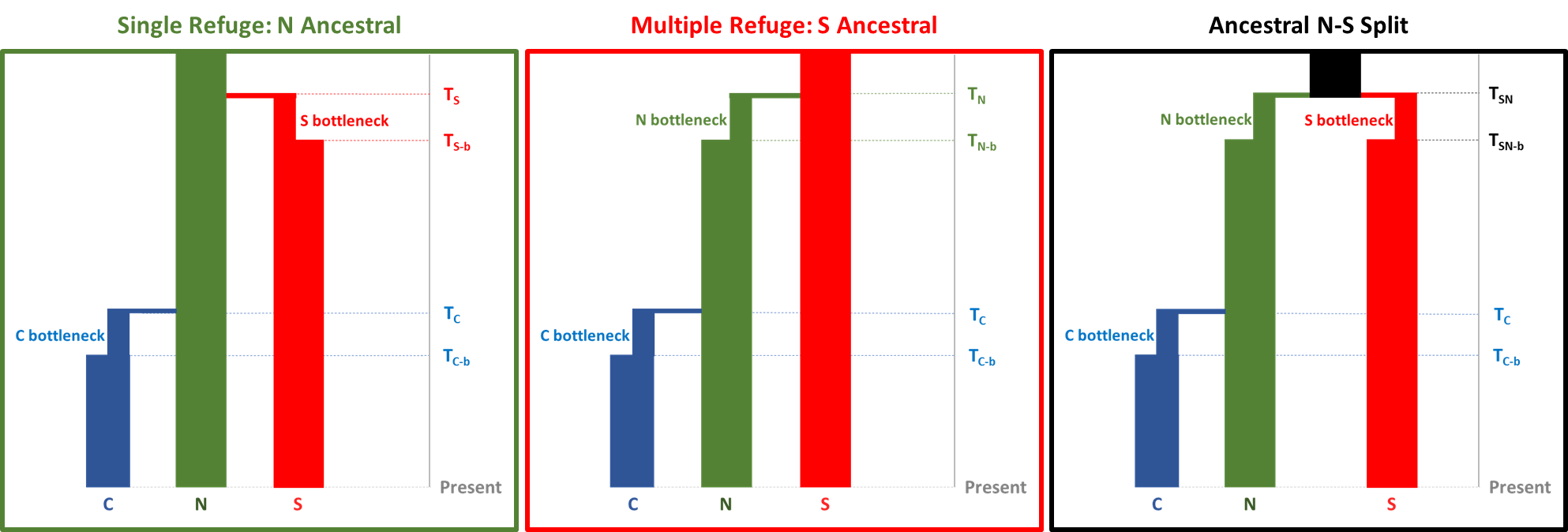


**Figure S4.5.** *Alternative scenarios in the second tier of ABC hypothesis testing.* All three of these scenarios involve the Central (C) population diverging from the Northern (N) population. In the refugial scenario (R3; left panel), first the Southern (S) cluster, then the Central cluster, diverged from the Northern cluster (i.e., the primary refugium). In the distributional shift scenario (DS1; middle panel), N diverged from S, and then C diverged from N in a stepping-stone fashion. The vicariance scenario (V; right panel) involves the separation of an ancestral population into S and N, followed by C diverging from N.

**Supplementary Results**

**Appendix S5.** Environmental factors used in species distribution models.

**Table S5.6.** *Correlations among environmental factors*. The table shows Pearson correlation coefficients among four environmental factors (MR) in each of four time periods: present-day, Mid-Holocene (MH), Last Glacial Maximum (LGM), and Last Interglacial (LIG).

| **Correlation of Environmental Factors** | | | | | | | | |
| --- | --- | --- | --- | --- | --- | --- | --- | --- |
| **Present** | | | |  | **MH** | | | |
|  | **MR1** | **MR2** | **MR3** |  |  | **MR1** | **MR2** | **MR3** |
| **MR2** | -0.29 |  |  |  | **MR2** | -0.28 |  |  |
| **MR3** | -0.55 | 0.04 |  |  | **MR3** | -0.03 | 0.24 |  |
| **MR4** | -0.82 | 0.38 | 0.60 |  | **MR4** | 0.34 | -0.14 | -0.61 |
| **LGM** | | | |  | **LIG** | | | |
|  | **MR1** | **MR2** | **MR3** |  |  | **MR1** | **MR2** | **MR3** |
| **MR2** | 0.13 |  |  |  | **MR2** | 0.36 |  |  |
| **MR3** | 0.30 | 0.39 |  |  | **MR3** | -0.49 | -0.16 |  |
| **MR4** | 0.70 | -0.28 | 0.04 |  | **MR4** | 0.88 | 0.41 | -0.72 |


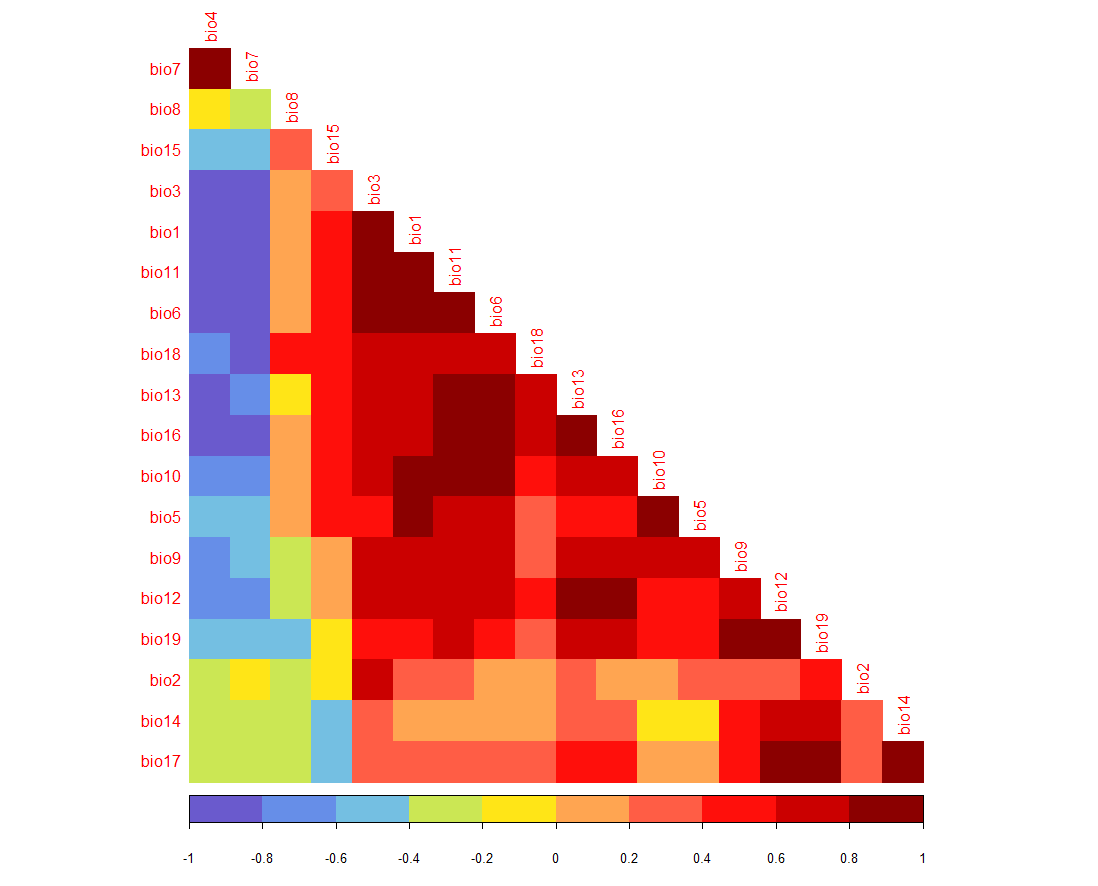


**Figure S5.6.** *Pearson correlation among 19 bioclimatic variables.* The plot of correlation coefficients (color-coded as a heat map, with strong positive correlation shown in red vs. negative in blue) among 19 bioclimatic (bio) variables, representing the “present” (1960–1990).


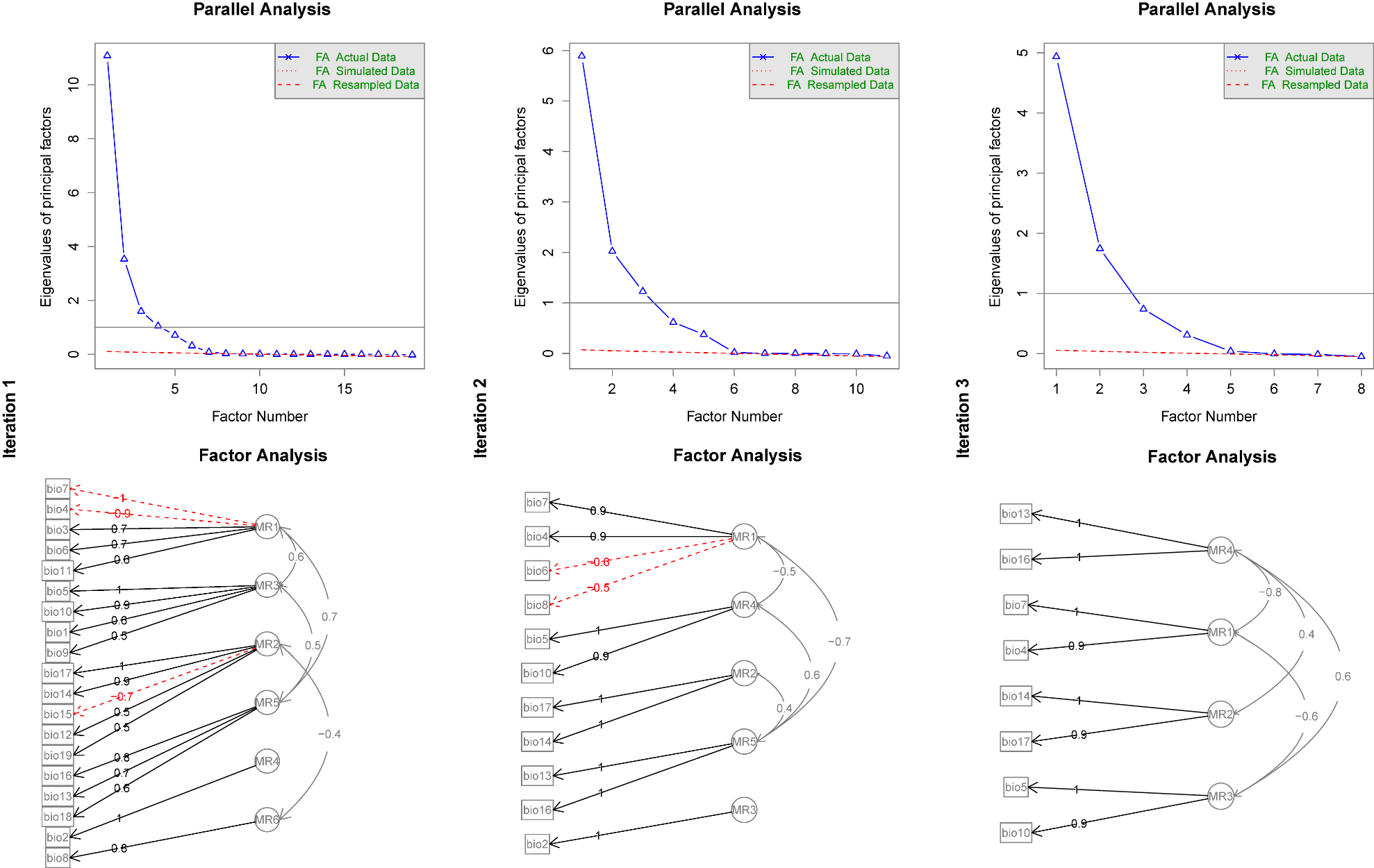


**Figure S5.7.** *Factor analysis.* The results shown here are for the present. Each column of panels represents one of three iterations of factor analysis. The top row depicts scree plots showing eigenvalues in descending order, the traditional threshold where eigenvalue = 1, and the confidence interval (red dotted lines) obtained via parallel analysis. The bottom row shows the factors and strength of correlation with the original bioclimatic variables. In the third and final iteration, abbreviations are as follows: MR1: temperature range; MR2: dry-season precipitation; MR3: summer temperature; MR4: wet-season precipitation.


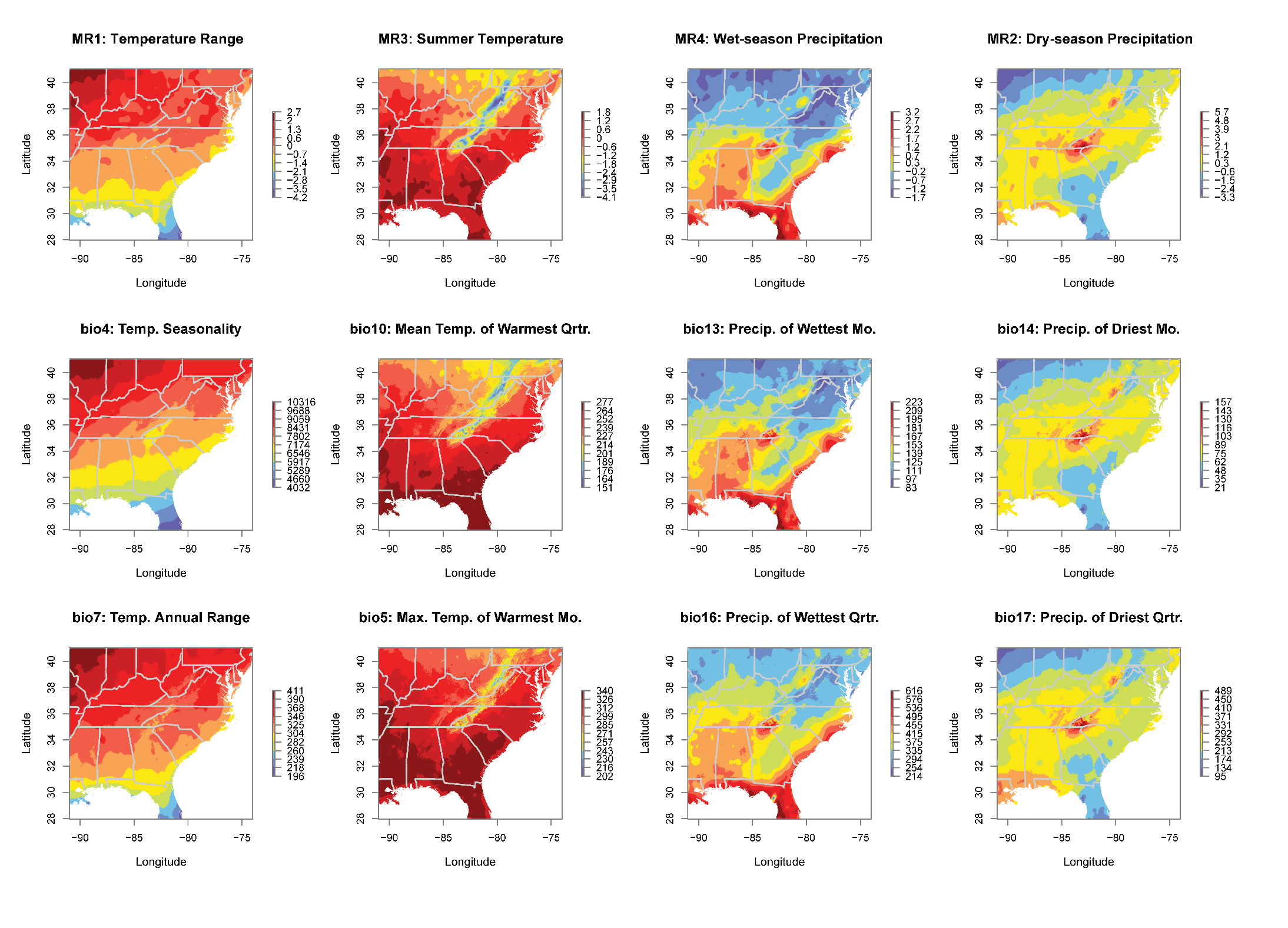


**Figure 3.** Distribution modeling of *R. flavipes* using four environmental factors. The four panels show habitat suitability or probability of occurrence (0 to 1) for the present as well as projections for the Mid-Holocene, Last Glacial Maximum, and the Last Inter-Glacial. **Correspondence with the reconstructed evolutionary history is highlighted.**

**Figure S5.8.** *Environmental factors and bioclimatic variables.* The top row of panels shows the four environmental factors obtained via factor analysis. In each column of panels, the top panel shows the factor that explains the variation in the original bioclimatic variables, whereas the middle and bottom panels show the bioclimatic variables that correlate most strongly with the factor in the top panel. The scales are different for each panel, but the colors go from dark blue (lowest value) to dark red (highest value). The environmental factors are unitless and go from negative to positive values. The unit for temperature variables is °C x 10. The unit for precipitation variables is mm.


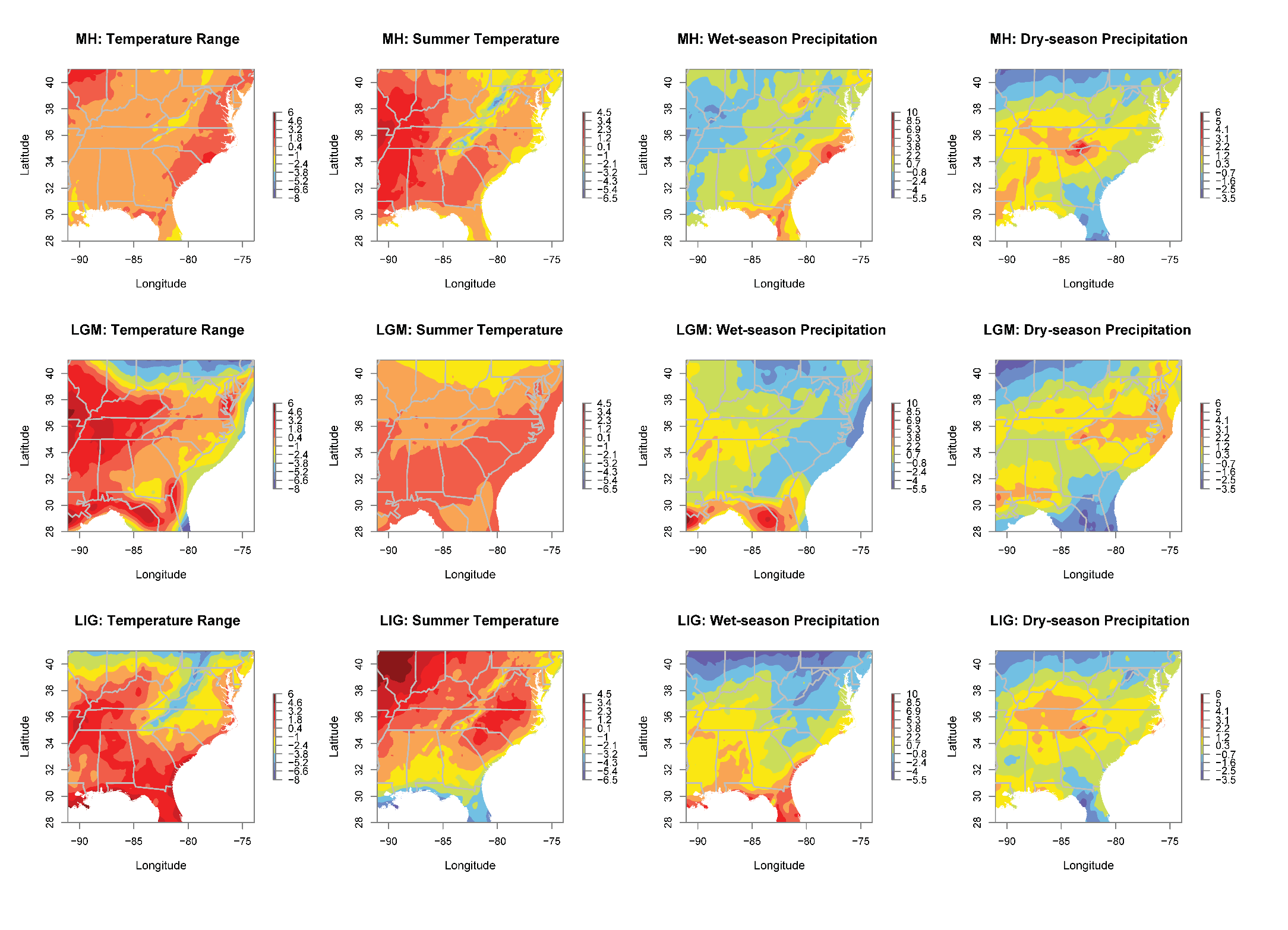


**Figure S5.9.** *Paleoclimatic factors.* Each column of panels shows one of the four environmental factors. The top row of panels depicts the four factors for the Mid-Holocene (MH), the middle and bottom rows shows the factors for the Last Glacial Maximum (LGM) and the Last Interglacial (LIG), respectively. The environmental factors are unitless and go from negative (dark blue) to positive (dark red) values.

**Appendix S6.** Genetic divergence, environment, and spatial structure.

To measure divergence among genetic populations, the following statistics were calculated: average number of nucleotide substitutions per site (Dxy; Nei 1987), net number of nucleotide substitutions per site (Da; Nei 1987), average number of pairwise nucleotide differences (Kxy; Tajima 1983), and *F*_ST_ (Hudson et al. 1992).

**Table S6.7.** *Genetic divergence.* Da = number of net nucleotide substitutions per site between populations; Dxy = average number of nucleotide substitutions per site between populations; Kxy = average number of pairwise nucleotide differences. Calculation of *F*_ST_ is based on Hudson et al. (1992), treating each polymorphic site as a separate locus. Pairwise comparisons were performed among the Northern (N), Central (C), and Southern (S) genetic clusters.

| **Locus** | **Comparison** | **Fixed Differences** | **Da** | **Dxy** | **Kxy** | ***F*_ST_** |
| --- | --- | --- | --- | --- | --- | --- |
| mtDNA | S-N | 9 | 0.013 | 0.026 | 28.611 | 0.494 |
|  | S-C | 15 | 0.018 | 0.028 | 31.066 | 0.659 |
|  | N-C | 3 | 0.005 | 0.012 | 13.690 | 0.447 |


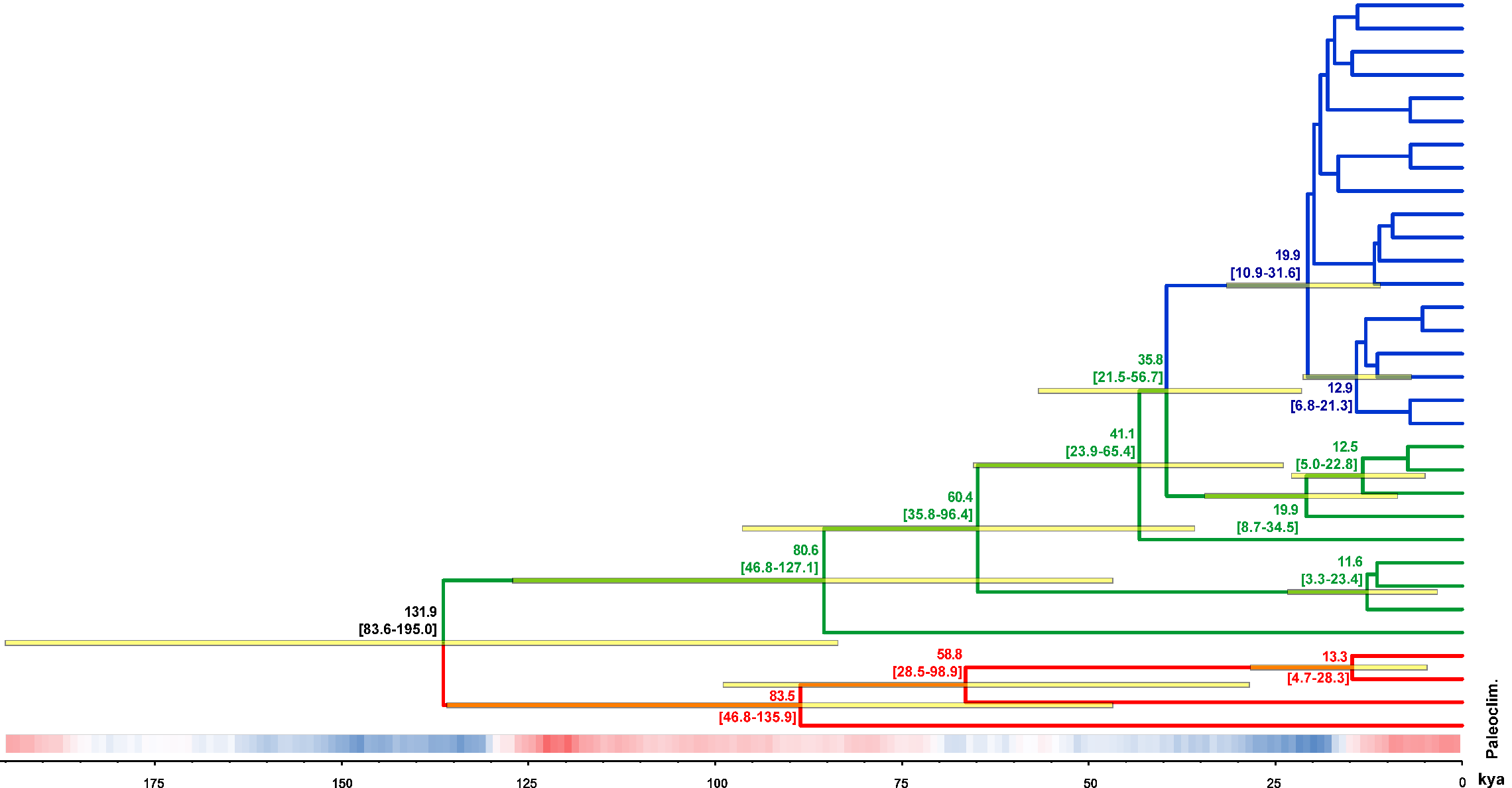


**Figure S6.10.** *MtDNA phylogeny with divergence times.* Median values of divergence times and 95% confidence intervals are shown at nodes, color coded by genetic cluster (red: southern; green: northern; blue: central). Bars at nodes represent 95% confidence intervals. Median divergence times below 10 kya (kya = 1,000 years ago) are not shown. Paleoclimate (global surface air temperature data from Hansen et al. 2013) is indicated by a bar at the bottom coded from blue (9.4°C) to red (15.6°C), representing cold to hot periods, respectively. The time scale is shown at the bottom.


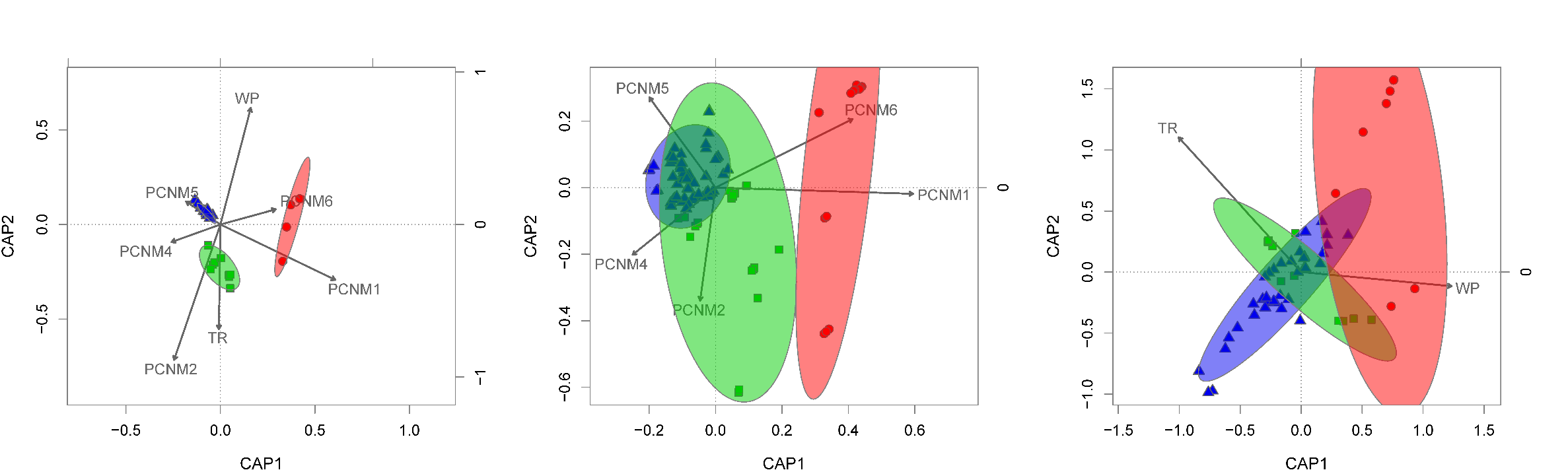


**Figure S6.11.** *Distance-based Redundancy Analysis (dbRDA).* The three panels show multivariate dbRDA-partitioned variation in the mtDNA sequence data explained by geography (eigenvectors obtained via Principal Coordinates analysis of Neighbor Matrices, PCNM) and the contemporary environmental data (factors obtained via factor analysis). The left panel shows the full model, the middle panel shows geography (eigenvectors with significant contribution to genetic variation: PCNM1, 2, 4, 5, and 6) after removing contributions of the environment, and the right panel shows the environment (factors with significant contribution to genetic variation: TR = “temperature range” and WP = “wet-season precipitation”) after factoring out geography. CAP stands for Constrained Analysis of Principal coordinates. CAP1 and CAP2 denote axes 1 and 2. The Northern cluster is shown in green, the Central in blue, and the Southern in red. The ellipses represent 95% confidence intervals.

**Appendix S7.** Phylogeographic hypothesis testing: error rates and parameter estimates.

**Table S7.8***. Type I and II error rates.* Type I (false positive) and type II (false negative) error rates for three alternative scenarios in the second tier of ABC hypothesis testing (see Fig. 6.11).

| **Scenario** | **Type I error rate** | **Type II error rate** |
| --- | --- | --- |
| **DS1** | 0.509 | 0.328 |
| **R3** | 0.446 | 0.354 |
| **V** | 0.734 | 0.159 |

**Table S7.9.** *Parameters of the best-fit scenario estimated using ABC.* N, C, and S represent the effective population sizes of the Northern, Central, and Southern clusters. T_N_ and T_C_ represent the time of divergence of the N and C clusters. b_N_ and b_C_ represent duration (number of generations) of bottleneck events. The parameters N_b_ and C_b_ represent effective population sizes during bottleneck events, and µ_mt_ and µ_nuc_ are mutation rates of the mtDNA and nDNA loci. Precision of parameter estimation is shown using the relative median of the absolute error (mean, median, and mode) for 500 data sets simulated using values drawn from posterior distributions.

| ***DS1: S-N;N-C*** | | | | | | |
| --- | --- | --- | --- | --- | --- | --- |
|  |  |  |  | ***Relative Median of the Absolute Error*** | | |
| *Parameter* | *Median* | *Quantile 2.5%* | *Quantile 97.5%* | *Mean* | *Median* | *Mode* |
| N | 82,700 | 32,300 | 213,000 | 0.326 | 0.324 | 0.374 |
| C | 1,170,000 | 516,000 | 4,530,000 | 0.581 | 0.546 | 0.624 |
| S | 174,000 | 63,100 | 245,000 | 0.226 | 0.227 | 0.309 |
| T_N_ | 64,800 | 26,400 | 115,000 | 0.270 | 0.264 | 0.351 |
| b_N_ | 5.68 | 1 | 10 | 0.398 | 0.426 | 0.750 |
| N_b_ | 4,980 | 559 | 44,700 | 1.096 | 0.865 | 0.905 |
| T_C_ | 8,630 | 2,750 | 22,500 | 0.390 | 0.362 | 0.438 |
| b_C_ | 8.52 | 1 | 10 | 0.352 | 0.366 | 0.666 |
| C_b_ | 168 | 101 | 4,570 | 7.410 | 2.409 | 0.453 |
| µ_mt_ | 1.21 x 10^-7^ | 3.33 x 10^-8^ | 4.13 x 10^-7^ | 0.411 | 0.422 | 0.477 |
| µ_nuc_ | 5.76 x 10^-9^ | 1.79 x 10^-9^ | 1.79 x 10^-8^ | 0.392 | 0.382 | 0.453 |

**Appendix S8.** Population size changes: standard and compound neutrality tests.

**Table S8.10***. Compound tests of neutrality in the Central cluster.* Both sampling site ID and genetic population membership of the out-group sequences used to perform the tests are shown. D = Tajima’s D; H = Fay and Wu's H; EW = Ewens and Watterson statistic; DH = Combination of D and H; HEW = Combination of H and EW; DHEW = Combination of D, H, and EW. Significant values are shown in bold. The statistics and p-values are reported in separate rows, which have been labeled accordingly. Note that there are no compound statistics, only p-values associated with the compound tests.

| Standard and Compound Neutrality Tests: Central Population | | | | | | | | |
| --- | --- | --- | --- | --- | --- | --- | --- | --- |
| Out-group:  Site | Out-group:  Cluster | D | H | EW | DH | HEW | DHEW |  |
| A70 | N | **-1.886** | 0.254 | 0.058 |  |  |  | *Statistic* |
|  |  | **0.015** | 0.431 | 1.000 | 0.237 | 1.000 | 1.000 | *p-value* |
| A106 | N | **-1.886** | 0.254 | 0.058 |  |  |  | *Statistic* |
|  |  | **0.014** | 0.430 | 1.000 | 0.237 | 1.000 | 1.000 | *p-value* |
| A142 | N | **-1.886** | -0. 531 | 0.058 |  |  |  | *statistic* |
|  |  | **0.014** | 0.184 | 1.000 | 0.083 | 1.000 | 1.000 | *p-value* |
| A60 | N | **-1.853** | -0.578 | 0.058 |  |  |  | *statistic* |
|  |  | **0.017** | 0.176 | 1.000 | 0.079 | 1.000 | 1.000 | *p-value* |

**Supplementary References**

Allouche O, Tsoar A, Kadmon R. 2006. Assessing the accuracy of species distribution models: prevalence, kappa and the true skill statistic (TSS). *J. Appl. Ecol.* 43:1223–1232.

Barbet-Massin M, Jiguet F, Albert CH, Thuiller W. 2012. Selecting pseudo-absences for species distribution models: how, where and how many? *Methods Ecol. Evol.* 3:327–338.

Beard RL. 1974. Termite biology and bait-block method of control. *Connecticut Agricultural Experimental Station Bulletin*. 748:1–9.

Beaumont MA, Zhang W, Balding DJ. 2002. Approximate Bayesian computation in population genetics. *Genetics* 162:2025–2035.

Breiman L. 2001. Random forests. *Machine Learning*. 45:5–32.

Buisson L, Thuiller W, Casajus N, Lek S, Grenouillet G. 2010. Uncertainty in ensemble forecasting of species distribution. *Glob. Change Biol.* 16:1145–1157.

Cattell RB. 1966. The scree test for the number of factors. *Multivariate Behav. Res*. 1:245–276.

Cornuet J-M, Pudlo P, Veyssier J, Dehne-Garcia A, Gautier M, Leblois R, Marin J-M, Estoup A. 2014. DIYABC v2.0: a software to make approximate Bayesian computation inferences about population history using single nucleotide polymorphism, DNA sequence and microsatellite data. *Bioinformatics* 30:1187–1189.

Cronbach LJ. 1951. Coefficient alpha and the internal structure of tests. *Psychometrika*. 16:297–334.

Feytaud J. 1920. Sur les jeunes colonies du termite lucifuge. *Comptes Rendus des Séances de l’Académie des Sciences*. 171:203–206.

Folmer O, Black M, Hoeh W, Lutz R, Vrijenhoek R. 1994. DNA primers for amplification of mitochondrial cytochrome c oxidase subunit I from diverse metazoan invertebrates. *Mol. Mar. Biol. Biotechnol.* 3:294–299.

Friedman JH. 2001. Greedy function approximation: a gradient boosting machine. *Ann. Stat.* 29:1189–1232.

Garrick RC, Collins BD, Yi RN, Dyer RJ, Hyseni C. 2015. Identification of eastern United States *Reticulitermes* termite species via PCR-RFLP, assessed using training and test data. *Insects* 6:524–537.

Hansen J, Sato M, Russell G, Kharecha P. 2013. Climate sensitivity, sea level and atmospheric carbon dioxide. *Phil. Trans. R. Soc. A* 371:20120294.

Harman HH, Jones WH. 1966. Factor analysis by minimizing residuals (minres). *Psychometrika*. 31:351–368.

Hijmans RJ, Cameron SE, Parra JL, Jones PG, Jarvis A. 2005. Very high resolution interpolated climate surfaces for global land areas. *Int. J. Climatol*. 25:1965–78.

Horn JL. 1965. A rationale and test for the number of factors in factor analysis. *Psychometrika.* 30:179–185.

Hudson RR, Slatkin M, Maddison WP. 1992. Estimation of levels of gene flow from DNA sequence data. *Genetics.* 132:583–589.

Nei M. 1987. Molecular Evolutionary Genetics. New York, USA: Columbia University Press.

Otto-Bliesner BL, Brady EC, Clauzet G, Tomas R, Levis S, Kothavala Z. 2006. Last Glacial Maximum and Holocene climate in CCSM3. *J. Clim.* 19:2526–2544.

Park YC, Maekawa K, Matsumoto T, Santoni R, Choe JC. 2004. Molecular phylogeny and biogeography of the Korean woodroaches *Cryptocercus* spp. *Mol. Phylogenet. Evol.* 30:450–464.

Phillips SJ, Anderson RP, Schapire RE. 2006. Maximum entropy modeling of species geographic distributions. *Ecol. Modell.* 190:231–259.

Revelle W. 2018. psych: procedures for personality and psychological research. R package version 1.8.4. Available from <https://CRAN.R-project.org/package=psych>.

Ripley BD. 1996. Pattern recognition and neural networks. Cambridge, UK: Cambridge University Press.

Simon C, Frati F, Beckenbach A, Crespi B, Liu H, Flook P. 1994. Evolution, weighting, and phylogenetic utility of mitochondrial gene sequences and a compilation of conserved polymerase chain reaction primers. *Ann. Entomol. Soc. Am.* 87:651–701.

Tajima F. 1983. Evolutionary relationship of DNA sequences in finite populations. *Genetics.* 105:437–460.

Thuiller W, Georges D, Engler R, Breiner F. 2016. biomod2: ensemble platform for species distribution modeling. R package version 3.3-7. Available from <https://CRAN.R-project.org/package=biomod2>.

Wang C, Zhou X, Li S, Schwinghammer M, Scharf ME, Buczkowski G, Bennett GW. 2009. Survey and identification of termites (Isoptera: Rhinotermitidae) in Indiana. *Ann. Entomol. Soc. Am.* 102:1029–1036.

Zeng K, Shi S, Wu C-I. 2007. Compound tests for the detection of hitchhiking under positive selection. *Mol. Biol. Evol.* 24:1898–1908.
